# Evidence-based clinical practice guidelines for nephrotic syndrome 2014

**DOI:** 10.1007/s10157-015-1216-x

**Published:** 2016-04-21

**Authors:** Shinichi Nishi, Yoshifumi Ubara, Yasunori Utsunomiya, Koichi Okada, Yoko Obata, Hiroyasu Kai, Hideyasu Kiyomoto, Shin Goto, Tsuneo Konta, Yoshie Sasatomi, Yoshinobu Sato, Tomoya Nishino, Kazuhiko Tsuruya, Kengo Furuichi, Junichi Hoshino, Yasuhiro Watanabe, Kenjiro Kimura, Seiichi Matsuo

**Affiliations:** Kobe University, Hyogo, Japan; Toranomon Hospital, Tokyo, Japan; Jikei University, Tokyo, Japan; Saitama Medical University, Saitama, Japan; Nagasaki University, Nagasaki, Japan; Tsukuba University, Ibaraki, Japan; Tohoku University, Miyagi, Japan; Niigata University, Niigata, Japan; Yamagata University, Yamagata, Japan; Fukuoka University, Fukuoka, Japan; Japan Community Health Care Organization Sendai Hospital, Miyagi, Japan; Kyushu University, Fukuoka, Japan; Kanazawa University, Ishikawa, Japan; Saitama Medical Hospital, Saitama, Japan; St. Marianna University, Kanagawa, Japan; Nagoya University, Aichi, Japan

## Preface

### 1. Background of this guideline

In Japan, original researches on nephrotic syndrome (NS) were initially performed by the Ministry of Health, Labour and Welfare (MHLW) NS research group. The first definition of NS was reported by the MHLW NS research group in 1973. Subsequently, the criteria for treatment effects were documented in 1974. Based on the continued clinical researches and social actions by the HLWM NS research group, the definition of refractory NS was determined in 1999. NS already treated with various agents, including steroids, that does not reach complete or incomplete remission within 6 months after the initiation of treatment is known as refractory NS.

In 2002, the HLWM NS research group published the “Guideline for Refractory Nephrotic Syndrome (Adult Cases).” This was the first NS guideline in Japan. Consequently, this group and the Japanese Society of Nephrology (JSN) published the second guideline, “Guideline for Nephrotic Syndrome,” in 2011. Currently, the collaborative working group of the MHLW and JSN aimed to publish and establish the third NS guideline in 2014. The new guideline aims to provide recommendations in clinical settings according to evidence-based medicine and it uses a description of clinical questions (CQs) according to the policy of publication for the clinical practice guidelines of the Medical Information Network Distribution Service (MINDS).

In 2012, an international guideline for glomerulonephritis, including NS, the “Guideline for Glomerulonephritis,” was published by the Kidney Disease Improving Global Outcome (KDIGO). Thus, the working group of the third NS guideline examined the contents of the KDIGO guideline as an important reference and re-evaluated Japanese treatment strategy in the past and the contents of previous guidelines already published in our country. We attempted that the third clinical guideline was considered to be appropriate for recent clinical practices for NS in Japan.

### 2. The Intended Purpose, Anticipated Users, and Predicted Social Significance of the Guidelines

The third NS guideline is intended as a reference for physicians engaging in the treatment of patients with NS. Practical clinical information on NS was included in this guideline for both specialists and nonspecialists of nephrology.

We described essential knowledge concerning NS in the first part and proposed many CQs associated with treatment in the later part. The response to each question was written as a statement with a recommendation grade. In the last part, we proposed a summary of a treatment strategy. In this summarized strategy, we proposed new treatment ideas based on previous ideas. The new strategy with algorithm figures may be helpful for the decision for treatment by physicians seeing nephrotic patients.

We found only limited articles on the treatments of adults with NS. The number of subjective patients was small in these articles. Therefore, the strategy addressed in this guideline did not absolutely force physicians to follow the stereotyped protocol, but rather we expected that our strategy would be helpful in decision making for the treatment of an individual patient with NS. Because aging patients with NS having various complications are increasing, the individual decision for the treatment of each patient is also necessary. We want to strongly insist that this guideline is not a decision basis for medical malpractice lawsuits or trials.

### 3. Patients within the scope of the guidelines

This guideline is intended as a reference for the treatment of patients with primary NS. In the preparation process of the guideline, we used evidence articles of pediatric patients if we could not find evidence articles of adult patients. In a part of the guideline, we referred to non-nephrotic cases. Recurrent NS occurring after kidney transplantation and NS associated with pregnancy were excluded from this guideline. For pregnant cases with NS, we hope that you refer to the “Clinical Guideline for Pregnancy of Kidney Disease Patients” that was edited by the JSN.

### 4. Preparation procedure

At first, we collected evidence articles available for guideline preparation. The working group of the NS guideline was set up. Nephrologists with sufficient knowledge and experience voluntarily participated in this working group.

On September 9, 2011, a progressive kidney disease research group supported by the MHLW research foundation, which acts to control refractory disease, opened the first collaborative meeting concerning 4 major nephrology diseases, including IgAN, NS, rapidly progressive glomerulonephritis, and polycystic kidney disease. Dr. Tsuguya Fukui, the president of St. Luke’s International Hospital, was invited as an adviser of this meeting. The members of the 4 working groups of the guideline learned the significant meaning of the guideline and the procedures for guideline preparation from his lecture. Thereafter, we began to write our guideline using common concepts.

Consequently, our working group of the NS guideline determined CQs with the Delphi method and free cross-talk communication. The survey of reference articles was performed using the PubMed database. For a basic survey, evidence articles were collected from already published papers until July 2012, and important articles were selected on demand from papers published after July 2012. Through several working group meetings and E-mail discussions, our working group summarized the contents of the NS guideline. In addition, several collaborative meetings concerning the 4 major kidney diseases, IgAN, NS, rapidly progressive glomerulonephritis, and polycystic kidney disease, were opened. In these meetings, the first CQs were properly revised. From August 2013 to October 2013, our working group asked for a review of the guideline by designated reviewers belonging to related academic societies. At the same time, we announced that we welcomed public comments from the members of the JSN. According to the suggestions from reviewers and public comments, we revised our guideline, established the final version, and publically answered the comments on the home page of the JSN.

### 5. Contents of the guideline

The contents of this guideline are related to those in Chapter 11 of the “2013 CKD Clinical Guideline Based on Evidence” and the guidelines for the 4 major kidney diseases, IgA nephropathy, NS, rapid progressive glomerulonephritis, and polycystic kidney, which were created based on research on progressive kidney diseases that was funded by scientific research aid from the MHLW.

### 6. Evidence levels and recommendation grades

Evidence was classified into 6 levels based on study design, and it was arranged roughly from the most reliable study type (Level 1) to the least reliable (Level 6). These levels do not necessarily represent rigorous scientific standards; they are intended for use as a convenient reference for quickly assessing the significance of various clinical data during the physician’s decision-making process.

- [Evidence Levels]
- Level 1: Systematic review/meta-analysis.
- Level 2: At least 1 randomized controlled trial (RCT).
- Level 3: A non-RCT.
- Level 4: An analytical epidemiologic study (cohort study or case–control study) or a single-arm intervention study (no controls).
- Level 5: A descriptive study (case report or case series).
- Level 6: Opinion of an expert committee or an individual expert, which is not based on patient data.

However, for systematic review/meta-analysis, the evidence level was decided based on the designs of underlying studies. If underlying study designs were mixed, the lowest level underlying the study was used to determine the overall evidence level. For example, meta-analysis of cohort studies would be Level 4, but the same Level 4 would also be assigned to meta-analysis including both RCTs and cohort studies.

In addition, a decision based on committee consensus was that all subanalyses and post hoc analyses of RCTs should be categorized at evidence Level 4. Accordingly, it was decided that the evidence level of findings representing the primary endpoints of a RCT would be Level 2, but that the evidence level of findings that were determined through subanalysis or post hoc analysis of that RCT would be Level 4.

When a statement related to a certain treatment was presented, consideration was given to the level of evidence serving as the basis of that statement, and a recommendation grade was assigned as follows:

- [Recommendation Grades]
- Grade A: Strongly recommended because the scientific basis is strong.
- Grade B: Recommended because there is some scientific basis.
- Grade C1: Recommended despite having only a weak scientific basis.
- Grade C2: Not recommended because there is only a weak scientific basis.
- Grade D: Not recommended because scientific evidence shows treatment to be ineffective or harmful.

If we found only a weak scientific basis for a certain statement concerning treatment, the members of the committee discussed the matter and decided on C1 or C2 for the recommendation grade. Thus, discrimination between C1 and C2 statements was based on expert consensus.

### 7. Issues on the preparation of this guideline

#### 1. Little evidence on Japanese patients

Compared with evidence articles regarding NS in foreign adult patients and Japanese children, evidence articles concerning Japanese adults with NS are less. Therefore, our statements were strongly affected by evidence from overseas countries and children with NS. It is doubtful whether the evidence from overseas country is suitable for Japanese nephrotic patients. Therefore, we paid careful attention to differences in the clinical status of NS between overseas countries and Japan. In Japan, observational and intervention studies of adults with NS have gradually progressed, and further active studies are expected in this field.

#### 2. Compatibility with the CKD clinical guideline and past NS guidelines

We paid careful attention to compatibility with the contents of Chapter 11 of the “2013 CKD Clinical Guideline.” There were no major conflict points between the current guideline and the past 2 guidelines, the “Guideline for Refractory Nephrotic Syndrome (Adult Cases)” and the “Guideline for Nephrotic Syndrome.” The current guideline was prepared according to the policy of the MINDS. The previous Japanese NS guidelines were not compliant with that policy. Therefore, some statements of the current guideline were distinct from the statements of previous guidelines. The statements and algorithm of this guideline were determined by mutual understanding of members belonging to the working group.

#### 3. Issues on medical resources

In general, the clinical guideline must consider medical resources associated with recommended statements. However, the current guideline did not discuss issues on medical cost; thus medical financial problems did not affect the contents of our guideline. In the next guideline, this point may be included.

#### 4. Guideline reflecting the opinions of patients

During the preparation processes of the clinical guideline, we needed to introduce the opinions of patients. However, this time, we unfortunately could not include the opinions of patients. We should refer to the opinions of patients in the next guideline, particularly in the case that the guideline is used for patients.

### 8. Financial sources and conflict of interest

All financial sources for this guideline were paid by the JSN and used for traffic fees, conference fees, etc. No payments were made to the members of the working group of this guideline.

All members of the working group of the guideline submitted documents for their conflicts of interest to the JSN. The submitted documents were kept with the JSN. We were asked to revise the guideline according to the suggestions from many reviewers from associated societies to avoid conflicts of interest. We asked for public comments from the members of the JSN. Finally, we revised this guideline referring to the suggestions from reviewers.

### 9. Publication and future revisions

#### 1. Public information on the guideline

This guideline was published in the Japanese version of the journal of the JSN and was concurrently released as a book in Japanese (by Tokyo Igakusha, Tokyo). This guideline was also uploaded to the homepage of the JSN. We hope this guideline will also be published on the MINDS website. Finally, we are planning to inform general physicians and medical staff regarding the contents of this guideline for the purpose of education them on the clinical strategy for NS.

#### 2. Practice and adherence to this guideline

We are planning to evaluate the states of practice and adherence to this guideline through a survey on the practical acts in the issue with grade B recommendation.

#### 3. Setting of necessary research themes in the future

From the statements with a C1 recommendation, we will choose new research questions and determine the necessary research themes in the CKD field. This point will be discussed in the Committee of CKD Action of the JSN. Active clinical research on the treatment strategy that focuses on Japanese adult patients with NS using approved immunosuppressive agents in our country are absolutely necessary because our country has approved only limited immunosuppressive agent use in the insurance system compared with overseas countries.

#### 4. Plan for revision

Revision of this guideline should be done 3 or 5 years later because new evidence is gradually increasing and new immunosuppressive agents are expected to be approved in the insurance system. At that time, we must document information from the perspective of patients and medical economy.

## I. Disease entity · definition (pathogenesis)

Nephrotic syndrome is a clinical syndrome showing specific features of heavy proteinuria and hypoalbuminemia or hypoproteinemia as its consequence. It is caused by increased permeability of serum protein through the damaged basement membrane in the renal glomerulus. The definition of nephrotic syndrome includes both massive proteinuria (≥3.5 g/day) and hypoalbuminemia (serum albumin ≤3.0 g/dL) (Tables 1, 4). Primary nephrotic syndrome has no background diseases, whereas secondary nephrotic syndrome has any background diseases. As a result of massive proteinuria and hypoalbuminemia, this syndrome is frequently accompanied by edema, dyslipidemia, abnormalities in coagulation/fibrinolysis, reduced renal function, and immunological disorders. The effect of treatment is determined by the urinary protein level after treatment (Tables 2, 3).

**Table 1:** Clinical definition of adult nephrotic syndrome

1. Proteinuria: ≥3.5 g/day and continuous (comparable to ≥3.5 g/gCr at spot urine)
2. Hypoalbuminemia: Serum albumin ≤ 3.0 g/dL
Serum total protein ≤ 6.0 g/dL is helpful
3. Edema
4. Dyslipidemia (Hyper LDL cholesterolemia)

The above urine protein and hypoalbuminemia are indispensable prerequisites for the clinical diagnosis of nephrotic syndrome

Edema is not an indispensable prerequisite but an important finding for nephrotic syndrome

Dyslipidemia is not an indispensable prerequisite for nephrotic syndrome

Oval fat body is helpful for diagnosis of nephrotic syndrome

**Table 2:** Therapeutic evaluation for nephrotic syndrome

The therapeutic evaluation is done by the amount of urine protein at 1 and 6 months after the initiation of treatment
Complete remission: urine protein <3.0 g/day
Incomplete remission I: 0.3 g/day ≤ urine protein <1.0 g/day
Incomplete remission II: 1.0 g/day ≤ urine protein <3.5 g/day
Non-response: urine protein ≥3.5 g/day

The diagnosis of nephrotic syndrome and therapeutic evaluation should be done by 24-hour urine collection. If to collect 24-hour urine is impossible, the ratio of urine protein and urine creatinine (g/gCr) at spot urine is available for the diagnosis of nephrotic syndrome and therapeutic evaluation

In principle, the evaluation of complete remission or incomplete remission at 6 months after the initiation of treatment includes the improvement of clinical finings and serum albumin

The evaluation of relapse is the condition that urine protein ≥ 1 g/gCr (1g/gCr) runs or ≥(2+) continues 2–3 times in a row

In Europe and the United States partial remission defines 50% or more of the reduction of urine protein, while the Japanese evaluation does not use this definition

**Table 3:** The classification by the response to treatment of nephrotic syndrome

Steroid resistant nephrotic syndrome: The enough dose of steroid treatment fails to achieve complete remission or incomplete remission I at 1 month after the initiation of treatment
Refractory nephrotic syndrome: The various treatments including steroid and immunosuppressive agents fail to achieve complete remission or incomplete remission I at 6 months after the initiation of treatment
Steroid dependent nephrotic syndrome: Steroid treatment is impossible to discontinue, because repeated over 2 times relapses appear after the reduction or discontinuation of steroid
Frequent relapse nephrotic syndrome: Over 2 times relapses appear in 6 months
Nephrotic syndrome requiring chronic treatment: Nephrotic syndrome to be treated by steroid or immunosuppressive agents over 2 years

**Table 4:** The definition of nephrotic syndrome in children

1. Nephrotic syndrome: Massive proteinuria (40 ≥ mg/h/m^2^) + hypoalbuminemia (serum albumin ≤ 2.5 g/dL)
2. Steroid sensitive nephrotic syndrome: Daily administrated prednisolone treatment attains the remission within 4 weeks
3. Relapse: After the remission urine protein of 40 ≥ mg/h/m^2^ or morning urine 100 mg/dL or more by dip stick continues for 3 days

## II. Diagnosis

### 1. Symptomatology · clinical condition

The predominant symptom of nephrotic syndrome is edema. In the early phase, edema appears in local parts such as the eyelids; in the advanced phase, generalized edema occurs with pleural effusion and ascites. Nephrotic syndrome is sometimes induced by upper respiratory infection or allergic reaction provoked by insect bites. It is important to evaluate the possibilities of secondary glomerular diseases in elderly patients with nephrotic syndrome.

### 2. Laboratory findings

Patients with nephrotic syndrome show various urinary abnormalities and renal dysfunction (Tables 5, 6). The degrees of proteinuria and hematuria differ with each histological type of nephrotic syndrome. High urinary specific gravity and various kinds of cast formation, including hyaline, granular, waxy, and fatty, are frequently noticed in nephrotic syndrome. Hematological abnormalities such as hypoalbuminemia, hypercholesterolemia, renal and liver dysfunction, electrolyte disorders, coagulation/fibrinolysis disorders, hormonal disorders, and anemia are usually found in patients with nephrotic syndrome.

**Table 5:** Examination findings of primary nephrotic syndrome

Examination	Measurement items	Major findings
Urinalysis	Urine volume, urine protein increase: urine protein, albuminemia (24-h collection or spot urine) fatty cast, oval fat body Fraction of urine protein Occult blood, urinary sediments Granular cast, waxy cast Selectivity of urine protein (clearance ration of IgG and transferrin)	Increase: urine protein, albuminemia fatty cast, oval fat body
Blood examination	Peripheral blood examination	Sometimes decrease: red blood cell, hemoglobin
	Biochemical examination	Decrease: total protein, albumin Sometimes decrease: Na, vitamin D, GFR Sometimes increase: BUN, Cr
	Lipid examination	Increase: total cholesterol, LDL, VLDL, La(a) ApoB, ApoC II, HDL-3 Stable: HDL Decrease: HDL-2
	Coagulation test	Increase: fibrinogen, FDP, D-dimer Decrease: antithrombin III, plasminogen
	Immunological test	Decrease: IgG and other immunoglobulins, complements
Chest X-ray	Cardiothoratic ratio, pulmonary vascular shadow cost-phrenic angle shadow of lung field	Sometimes: pulmonary congestion
Ultrasonography	Deep vein thrombosis in lower extremities	Collapse of venous system due to decrease of circular blood volume
Renal biopsy	Light microscopy Immunofluorescence microscopy	The definitive diagnosis is usually determined electron microscopy by renal biopsy

When secondary nephrotic syndrome is suspected from patient’s conditions, the examinations according to each baseline disease should be added. (For example; In the case of lupus nephritis, the examinations concerning collagen diseases should be done as additional items.)

**Table 6:** Examination findings of secondary nephrotic syndrome

Examination	Measurement items	Major findings
Urinalysis	Occult blood Urine Bence Jones protein	Positive in purpura nephritis or vasculitis positive in paraproteinemia
Blood examination	Peripheral blood examination	Pancytopenia or hemolytic anemia in lupus nephritis Leukocytosis and thrombocytosis in the cases with infectious diseases ad vasculitis
	Biochemical examination	Blood sugar markers such as blood glucose, HbA1c, and glycoalbumin in diabetic nephropathy CRP and inflammatory reactions increase in vasculitis and purpura nephritis Paraprotein or cryoglobumin is confirmed in in the cases with paraproteinemia
	Lipid examination	The abnormality of IDL or ApoE is confirmed in lipoprotein glomerulopathy
	Immunological examination	Anti-nuclear antibody , anti-ds-DNA antibdy, anti-Sm antibody, anti-phosphlipid antibody increase and complements decrease in lupus nephritis The positive findings are confirmed in bacterial culture and antigen/antibody detection for pathogenic microbes
Renal biopsy		The specific findings are observed in each secondary disease, thus the renal biopsy is useful for the definitive diagnosis of secondary diseases
Imaging test		Neoplastic diseases are diagnosed by various imaging tests such as CT, MRI, ultrasonography and bone marrow aspiration
Genetic test		Genetic tests are useful in the genetic illnesses

## III. Epidemiology · prognosis

### 1. Incidence · prevalence · recurrence rate

The researchers of the Committee for the Standardization of Renal Pathological Diagnosis and the Working Group for the Renal Biopsy Database of the Japanese Society of Nephrology had set up the J-RBR/J-KDR (Japan Renal Biopsy and Kidney Disease Registry) since 2007, and the epidemiology of nephrotic syndrome in Japan was gradually revealed. In the analysis of cases registered to the J-RBR until the end of 2010, primary glomerular disease was the most frequently occurring glomerular disease and diabetic nephropathy was the most frequent among the secondary glomerular diseases. The total cases of membranous nephropathy (MN) and minimal change nephrotic syndrome (MCNS) were close to 80 % among the primary glomerular diseases. In the analysis of nephrotic syndrome patients aged ≥65 years, the ratios of diabetic nephropathy and amyloid nephropathy were highest, next to primary glomerular disease.

MCNS, focal segmental glomerulosclerosis (FSGS), MN, and membranoproliferative glomerulonephritis are known to relapse frequently. However, a wide range of relapse rates was reported in previous articles; thus, prospective follow-up surveys such as the Japanese Nephrotic Syndrome Cohort Study (JNSCS) are expected to provide precise rates.

### 2. Remission rate · nonresponsive rate · renal prognosis

Remission rates, nonresponsive rates, and prognosis vary across the histological types of nephrotic syndrome. MCNS shows a higher remission rate of ≥90 %, whereas the recurrence rate is also higher at 30–70 %. Compared with MCNS, FSGS shows a lower remission rate and poorer renal prognosis resulting in end-stage renal disease. About half of the cases of FSGS are nonresponders to steroid treatment. The responsive rates and renal prognosis vary across the variant types of FSGS. In the data in Japan, the renal survival rate was 33.5 % at the 20-year follow-up examination. MN showed a high remission rate in Japanese patients. Complete or incomplete remission by single steroid treatment was achieved in 73.1 %. Approximately 30 % of cases showed spontaneous remission. However, the renal survival rate was 59 % at the 20-year follow-up examination.

### 3. Incidence of complications

Various complications develop in patients with nephrotic syndrome. Although cohort studies performed abroad revealed a high incidence of cardiovascular events, the actual state in Japan seems to be different. Treatment with glucocorticoids and/or immunosuppressants, and nephrotic syndrome itself, often make patients susceptible to infection, the true rate of which remains to be determined. Reports from abroad also highlighted a high incidence of thromboembolic events. Furthermore, the westernized lifestyle makes the Japanese population more susceptible to thrombosis and therefore should receive research attention. Malignant tumors have been considered a common complication in patients with nephrotic syndrome. However, according to recent surveys, the co-occurrence rate of malignant tumors with nephrotic syndrome seems relatively low in Asian countries such as Japan and China compared with that in Western countries. Acute renal failure is another representative complication in patients with nephrotic syndrome, especially in the elderly.

## IV. Treatment

### 1. Clinical Questions for Treatment

#### 1. Minimal change nephrotic syndrome and focal segmental glomerulosclerosis


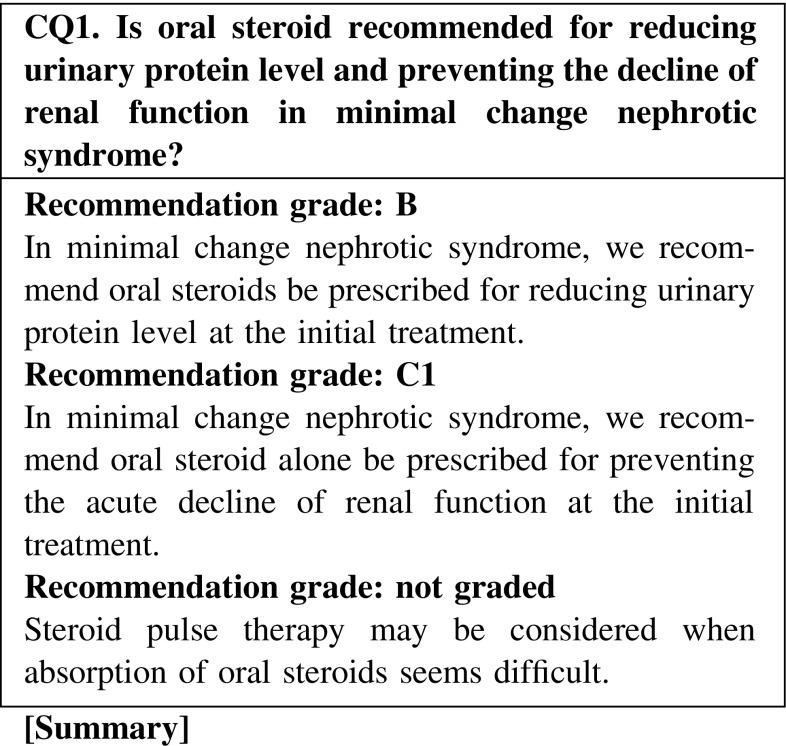


Oral steroid therapy is usually administered as the initial treatment for minimal change nephrotic syndrome. In the evaluation of efficacy, a high response rate of ≥90 % was found. Steroid pulse therapy may be considered when absorption of oral steroids seems difficult because of intestinal edema, diarrhea, and other conditions.
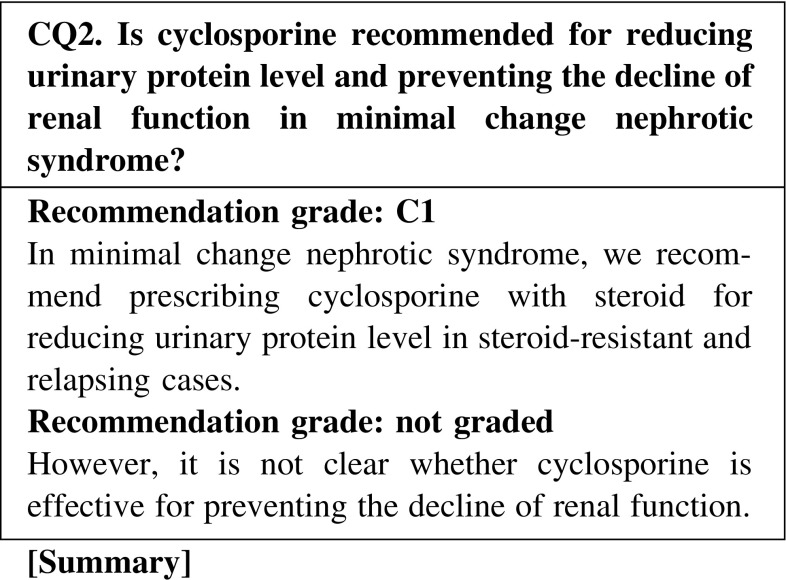


Compared to steroid alone treatment, the combination treatment of cyclosporine and steroid is effective for reducing urinary protein level and shortening the duration of achieving remission in relapsing cases of minimal change nephrotic syndrome. However, it is not clear whether cyclosporine is effective for preventing the decline of renal function.
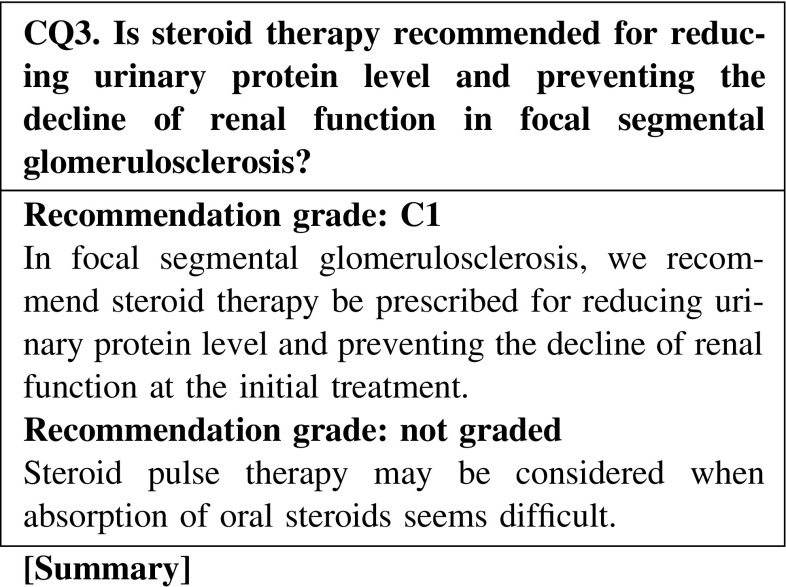


Oral steroid therapy as an initial treatment is effective for focal segmental glomerulosclerosis, showing a remission induction rate of 20–50 %. However, the efficacy of steroids varies depending on histological variants. The concomitant use of immunosuppressants is necessary for steroid-resistant cases.
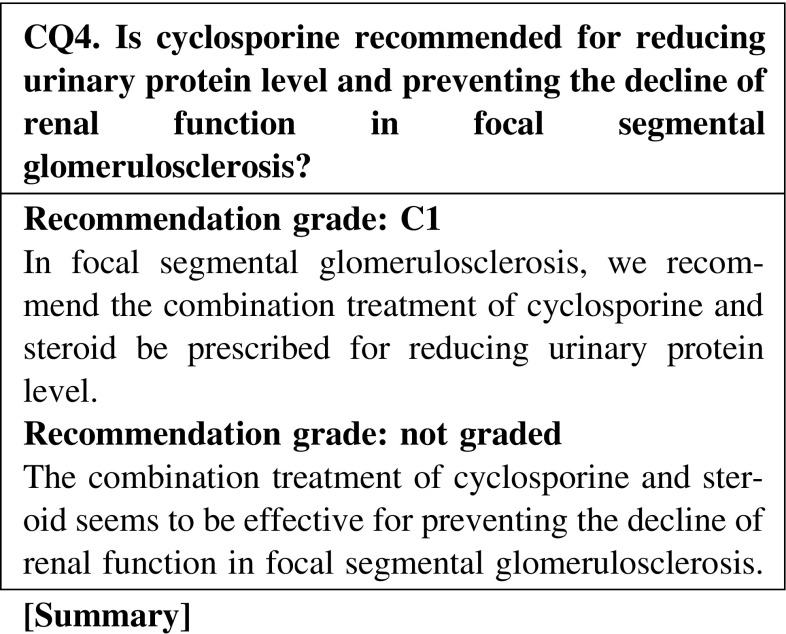


The combination treatment of cyclosporine and steroid is effective for inducing remission in focal segmental glomerulosclerosis. Evidence showing that the combination treatment of cyclosporine and steroid is effective for preventing the decline renal function is limited; however, some extent of efficacy is expected. The possibilities of cyclosporine nephrotoxicity with the long-term use of the drug are unclear.
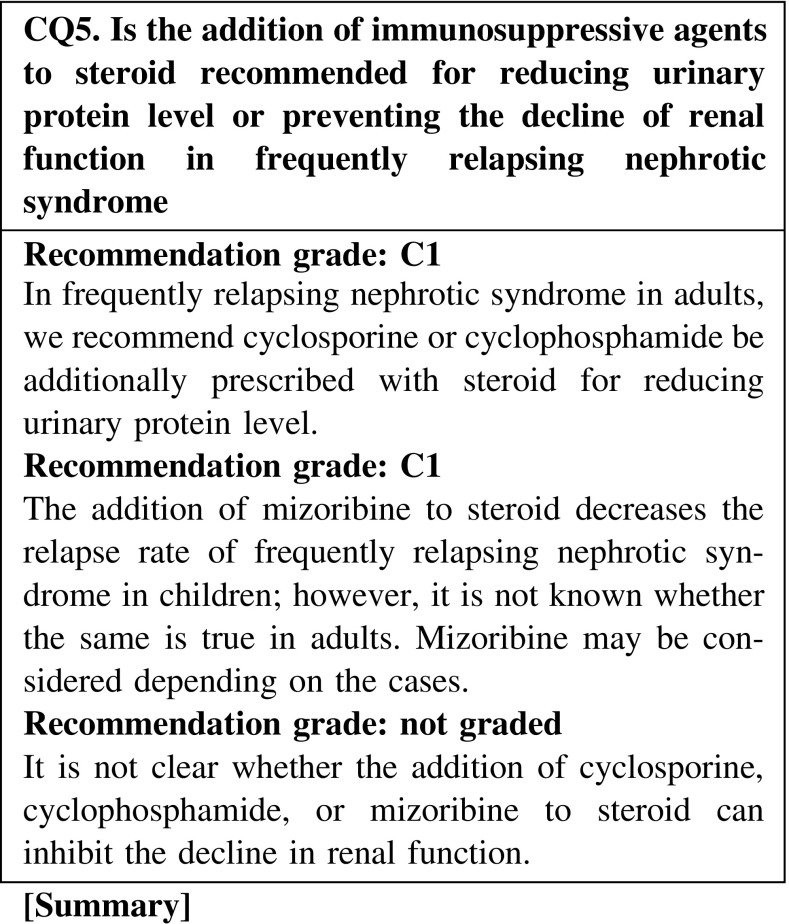


The addition of oral cyclosporine or cyclophosphamide to steroid is effective for the reduction of urinary protein level in frequently relapsing nephrotic syndrome in adults. However, the efficacy of mizoribine is unknown. Although renal function might be preserved by maintaining complete remission, there is no clear evidence indicating that these additional immunosuppressive agents are effective for preventing the decline of renal function.
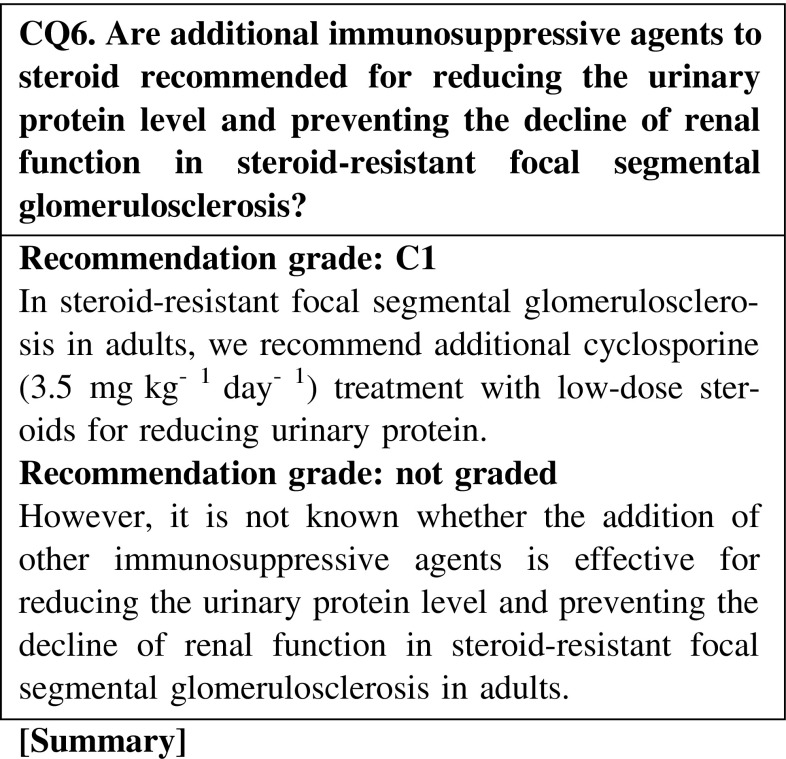


The addition of cyclosporine is effective for reducing the urinary protein level in steroid-resistant focal segmental glomerulosclerosis in adults. Maintaining the remission of nephrotic syndrome is associated with preventing the decline of renal function. However, the addition of chlorambucil and mycophenolate mofetil is not superior to that of cyclosporine for reducing urinary protein level. There are no sufficient data indicating that these immunosuppressive agents have direct renoprotective effects in adult cases of steroid-resistant focal segmental glomerulosclerosis.

#### 2. Membranous nephropathy


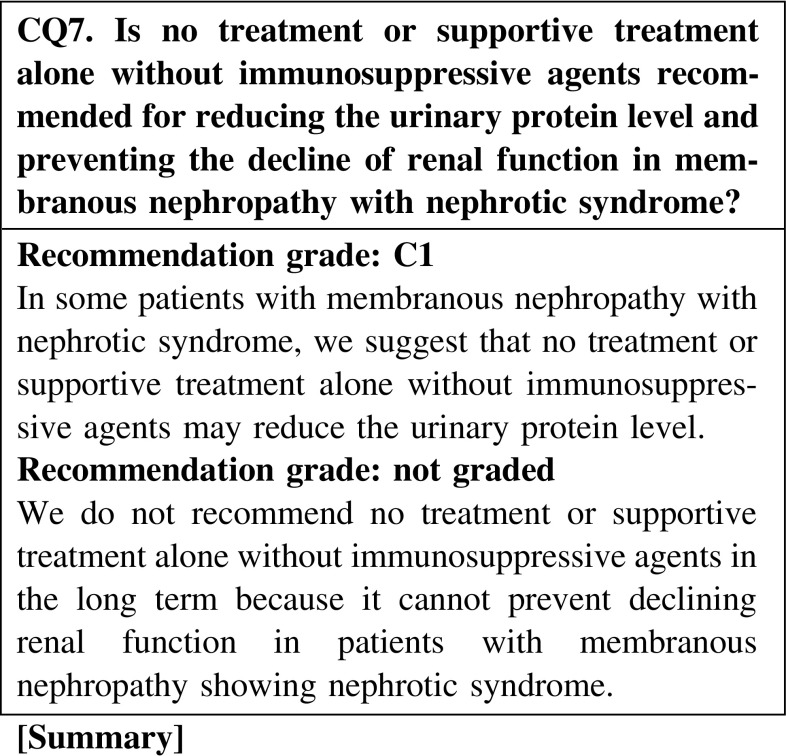


No treatment or supportive therapy alone without immunosuppressive agents is effective for reducing the urinary protein level in some patients with membranous nephropathy showing nephrotic syndrome; however, these are not expected to prevent the decline of renal function. In particular, this type treatment may worsen the renal prognosis of patients with severe urinary protein excretion.
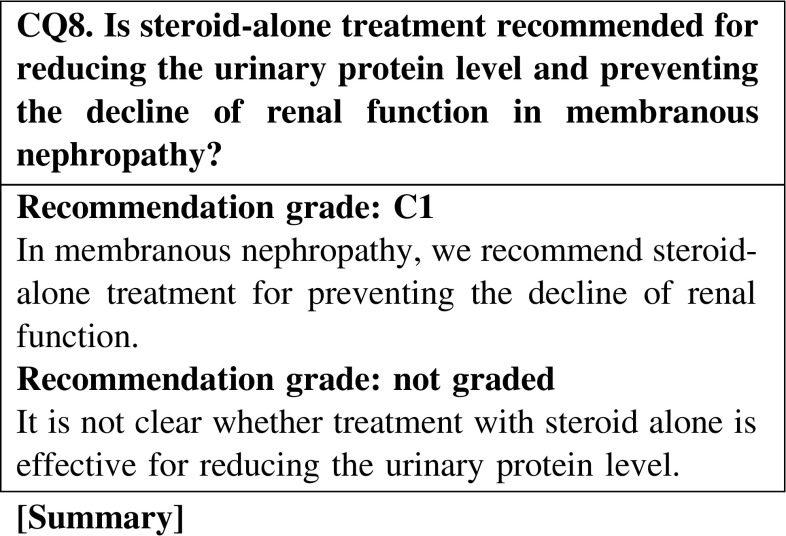


Compared with no treatment, steroid-alone treatment is not effective for reducing the urinary protein level in membranous nephropathy. In a retrospective study in Japanese patients with membranous nephropathy, the remission rates did not show any significant differences between three treatment groups (steroid alone, steroid and cyclophosphamide, and supportive treatment); however, treatment with steroid alone and the combination of steroid and cyclophosphamide showed significant effectiveness in preventing the decline of renal function compared with supportive treatment.
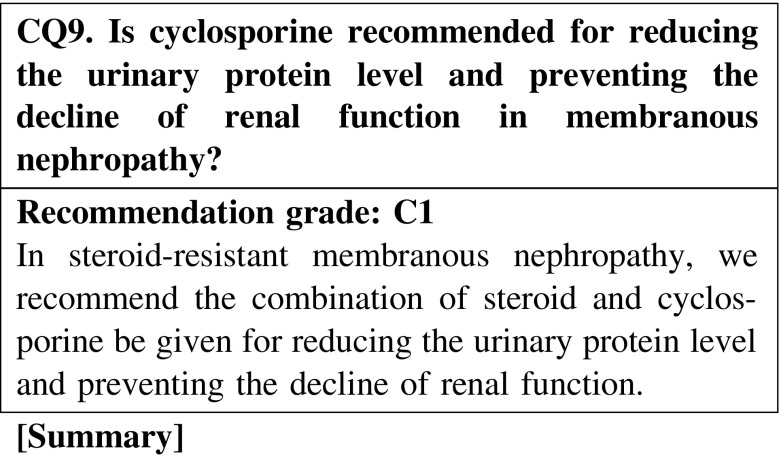


The combination treatment with steroid and cyclosporine is effective for reducing the urinary protein level and preventing the decline of renal function compared with treatment with steroid alone. Between steroid with cyclosporine and steroid with alkylating agents, the superiority of treatment with steroid and cyclosporine has not been recognized.
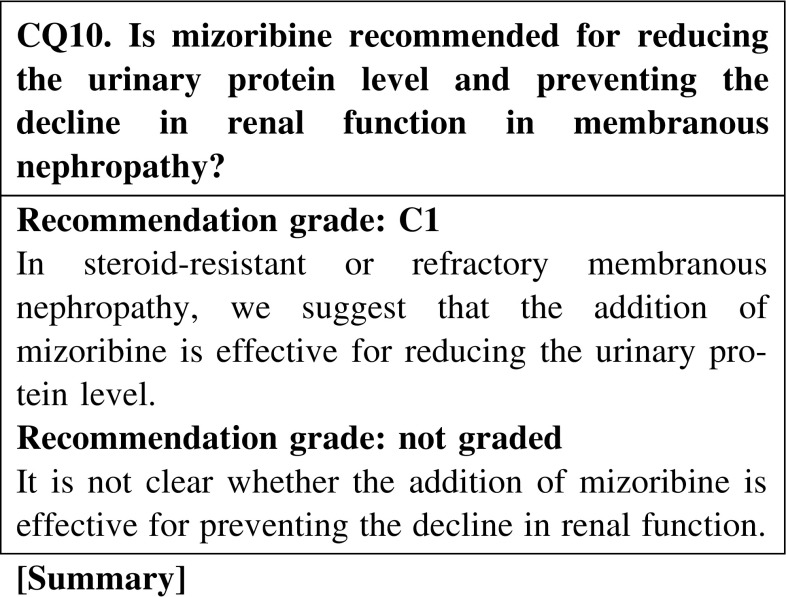


It has been reported that the addition of mizoribine to steroid reduces the urinary protein level in patients with membranous nephropathy. However, this effect of mizoribine has not been confirmed in appropriately sized randomized control trials. The dose of mizoribine should be carefully reduced in patients with chronic renal failure.
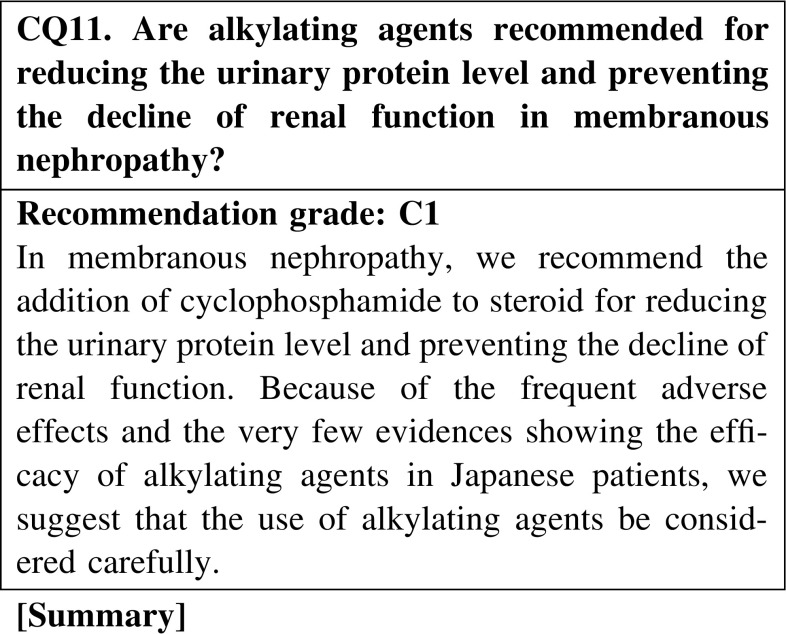


In overseas countries, it is generally accepted that the combination treatment with steroid and alkylating agents is superior to steroid-alone treatment for inducing the remission of nephrotic syndrome in membranous nephropathy. Although the study is retrospective, the results suggest that the efficacy of steroid-alone treatment is similar to that of the combination treatment with steroid and alkylating agents in Japanese patients. Attention should be given to the high frequency of adverse effects of alkylating agents. Cyclophosphamide has fewer adverse effects than chlorambucil.
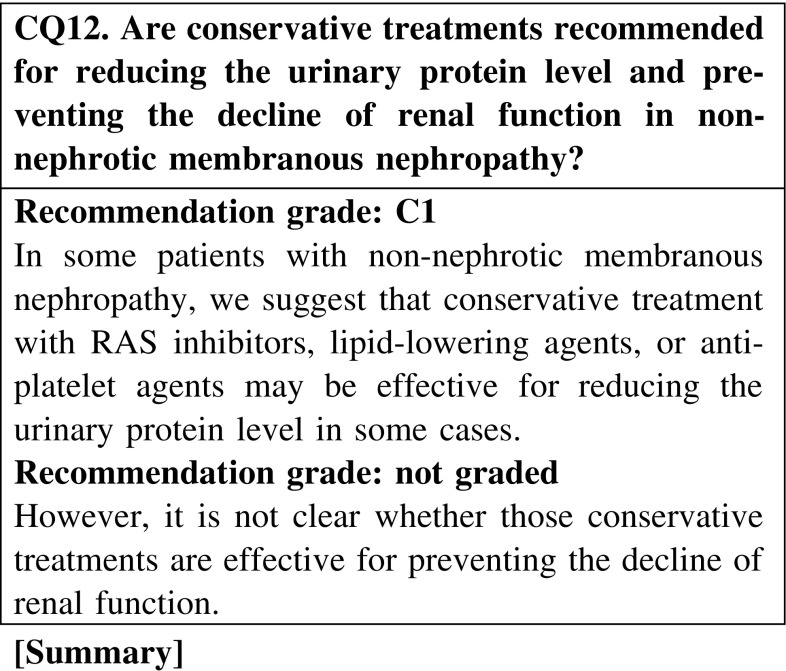


Conservative therapies with RAS inhibitors, lipid-lowering agents, or antiplatelet agents are effective for reducing the urinary protein level in some patients with membranous nephropathy accompanied by a non-nephrotic rage of proteinuria. However, these conservative treatments are not expected to prevent the decline of renal function.

#### 3. Membranoproliferative glomerulonephritis


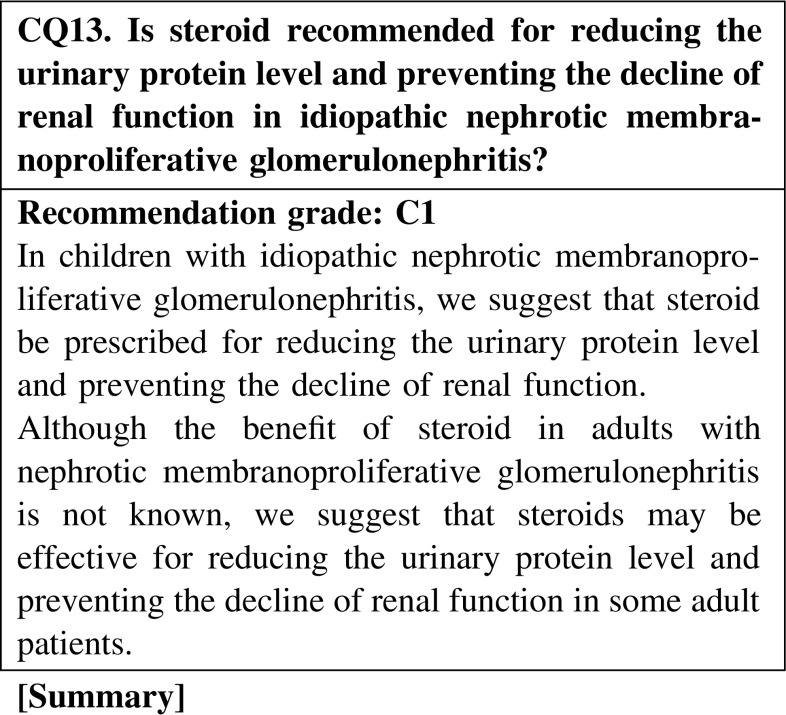


Observational studies suggest that steroid is beneficial for reducing the urinary protein level and preventing the decline of renal function in children with idiopathic membranoproliferative glomerulonephritis. Although evidences concerning the treatment of adult patients with idiopathic membranoproliferative glomerulonephritis are inconsistent, we suggest that steroid is the acceptable treatment agent in some adult patients with idiopathic membranoproliferative glomerulonephritis.

#### 4. How to use steroids


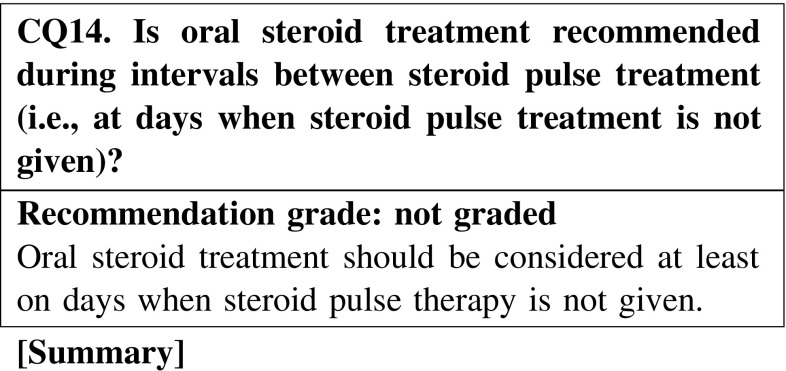


The half-life of methylprednisolone is short, i.e., 1–3 h, whereas that of oral steroids is long, i.e., 12–36 h. Therefore, oral steroid treatment is considered necessary on days when steroid pulse therapy is not given.
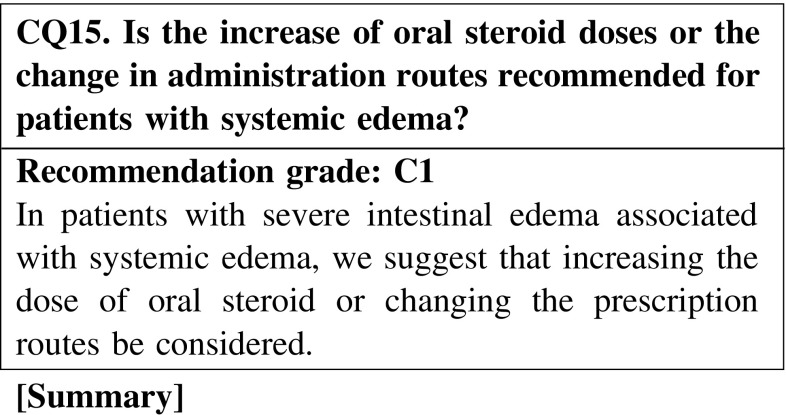


The efficacy of oral steroid seems to be diminished in patients with systemic edema. Therefore, it may be necessary to consider intravenous steroid therapy or steroid pulse therapy in patients with systemic edema.
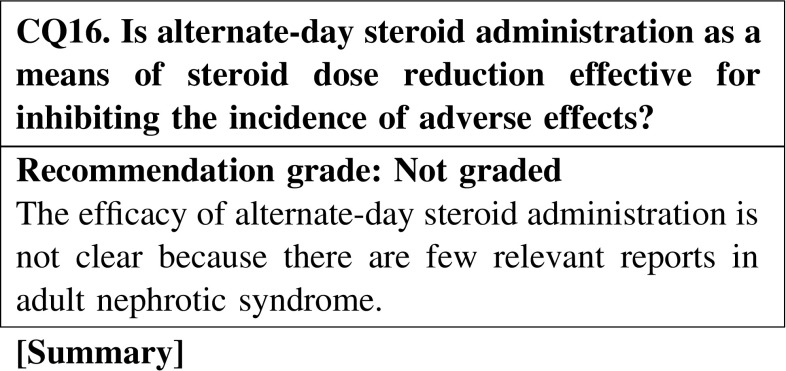


Limited evidence exists on whether alternate-day steroid treatment for nephritis as a means of dose reduction is effective for inhibiting adverse reactions. Further studies are warranted.
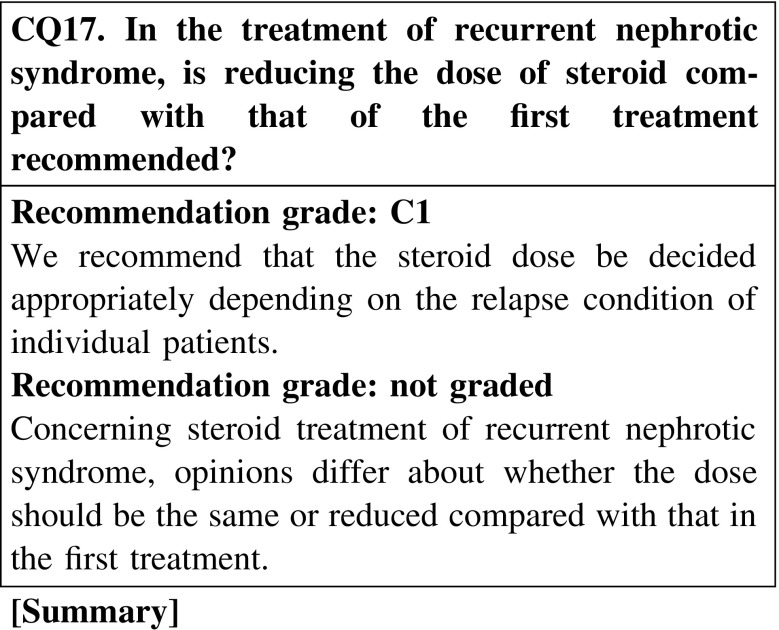


In steroid treatment of recurrent nephrotic syndrome, opinions differ about whether the treatment should be different from the initial treatment. There are two conflicting opinions: (i) recurrent nephrotic syndrome should be treated in the same way as the initial treatment, and (ii) recurrent nephrotic syndrome should be treated with prednisolone at a dose of 20–30 mg/day. No consensus has been reached.
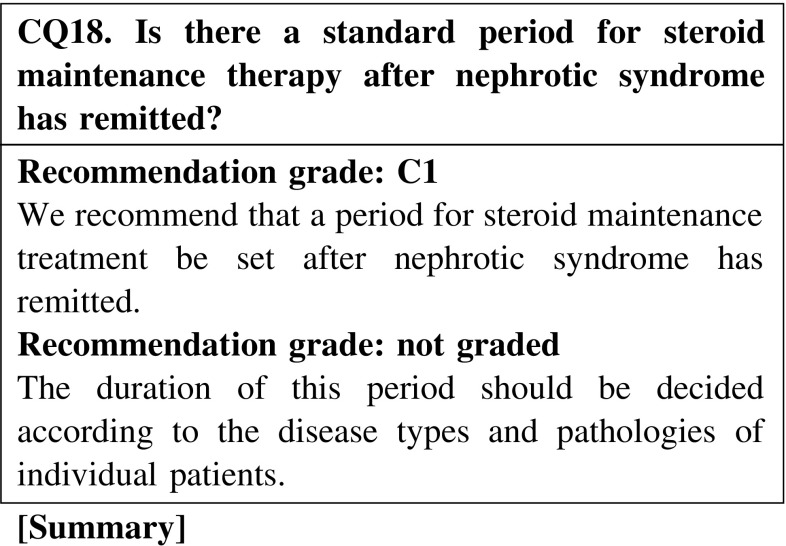


There is no clear evidence suggesting a standard period for steroid maintenance therapy after nephrotic syndrome has remitted.

#### 5. Immunosuppressive agents not allowed by medical insurance (at the time of description of this guideline in 2013)


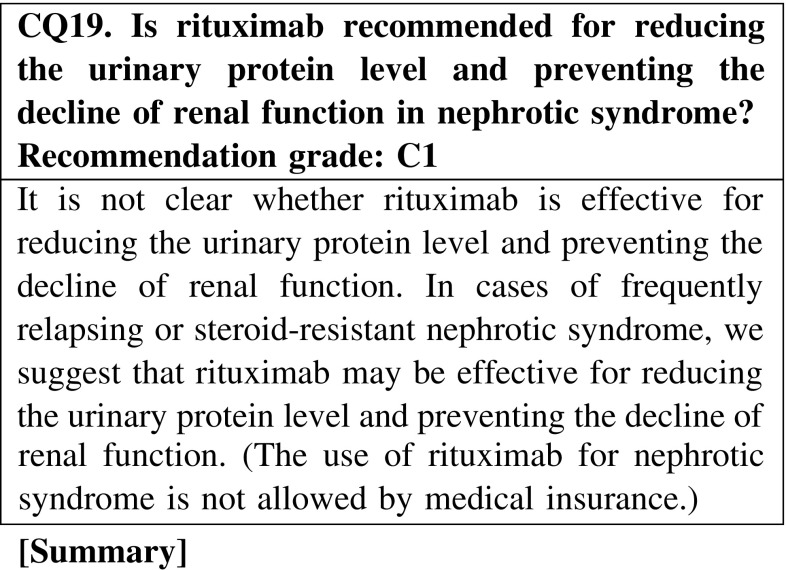


Rituximab may be effective for reducing the urinary protein level in nephrotic syndrome; however, clinical studies are rare in adult cases. Rituximab could be an option for the treatment of nephrotic syndrome, but we cannot conclude that it is an effective agent.
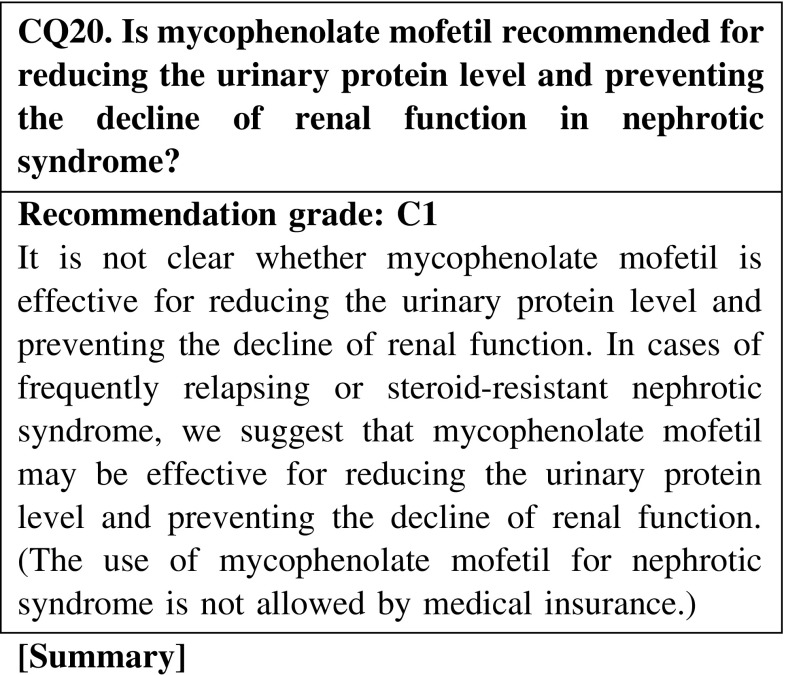


Mycophenolate mofetil may be effective for reducing the urinary protein level in nephrotic syndrome; however, clinical studies are rare in adult patients with nephrotic syndrome. Mycophenolate mofetil could be an option for the treatment of nephrotic syndrome, but we cannot conclude that it is an effective agent.
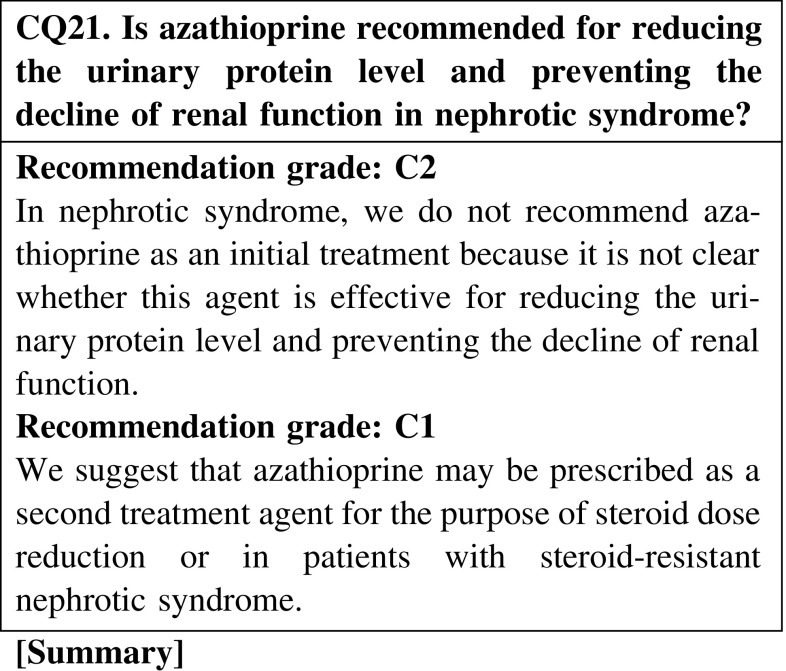


Azathioprine may be effective for reducing the urinary protein level in nephrotic syndrome; however, clinical studies of adult cases of nephrotic syndrome are rare. Azathioprine could be an option for the treatment of primary nephrotic syndrome, but we cannot conclude that it is an effective agent. We do not recommend this agent for initial treatment.

#### 6. Nephrotic syndrome in the elderly


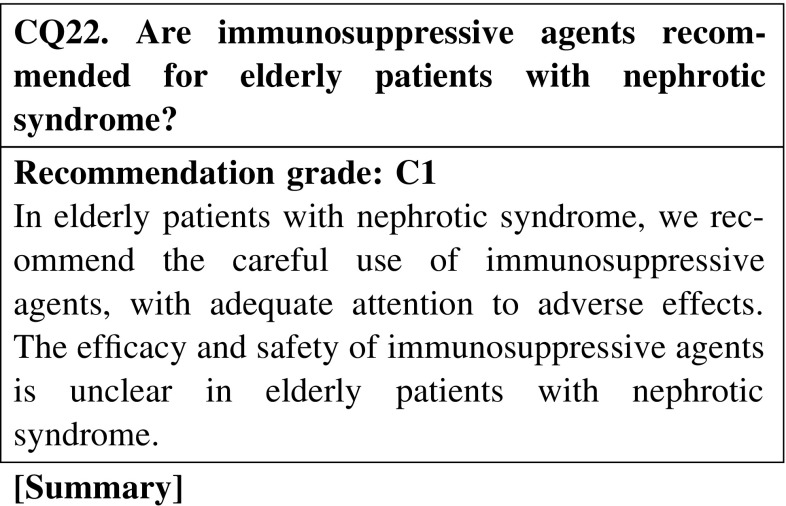


Few clinical studies have evaluated the efficacy of immunosuppressive agents in elderly patients with nephrotic syndrome; however, the efficacy for reducing the urine protein level was reported to be similar to that in younger patients. In contrast, the incidence rate of adverse effects in elderly patients is higher than that in younger patients. The incidence rate of adverse effects of chlorambucil is higher than that of cyclophosphamide.

#### 7. Adjunctive and supportive treatments


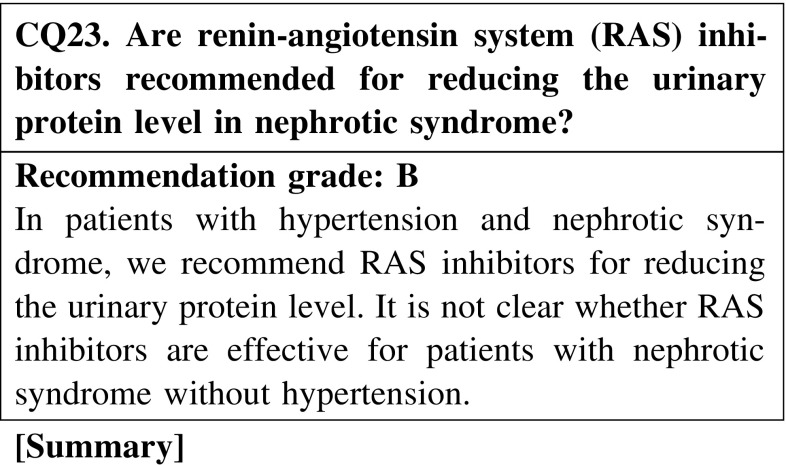


A number of studies have shown that RAS inhibitors reduce the urinary protein level in patients with membranous nephropathy, membranoproliferative glomerulonephritis, and focal segmental glomerulosclerosis with nephrotic syndrome; however, complete remission by RAS inhibitors alone has been seldom reported. Furthermore, very little is known about the effect of RAS inhibitors in patients with nephrotic syndrome without hypertension.
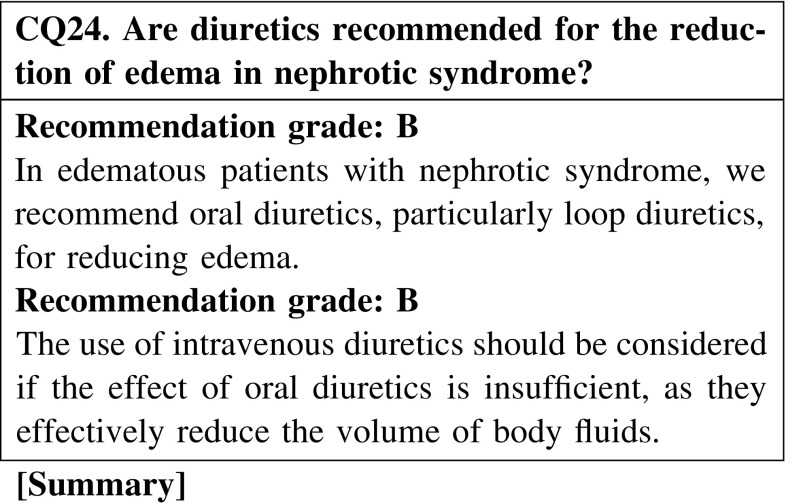


Oral loop diuretic monotherapy or oral loop diuretics combined with thiazide diuretics are effective for edema reduction in patients with nephrotic syndrome. Intravenous loop diuretics are considered appropriate for patients with severe edema. No study has compared the effects of single injection, multiple injection, and continuous injection.
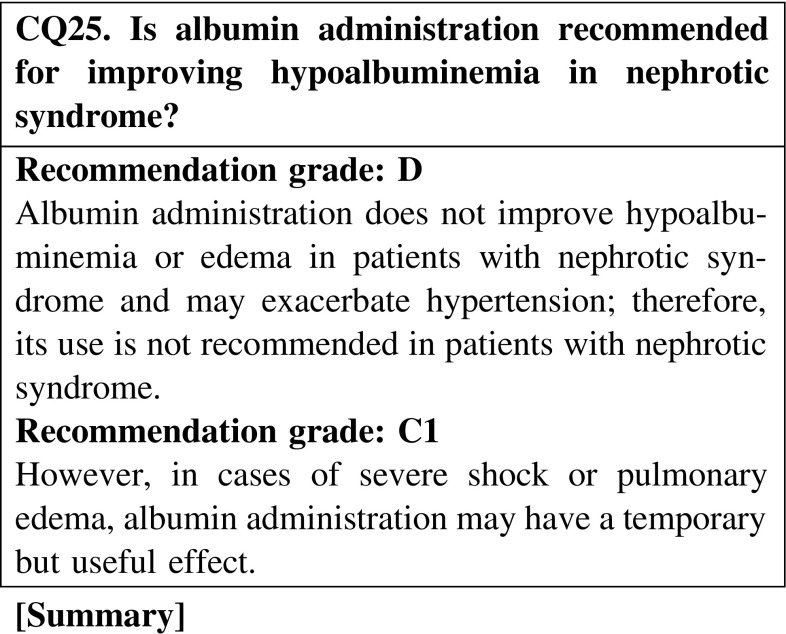


It is not clear whether albumin administration improves edema or has a diuretic effect in patients with nephrotic syndrome. Rather, it may exacerbate hypertension.
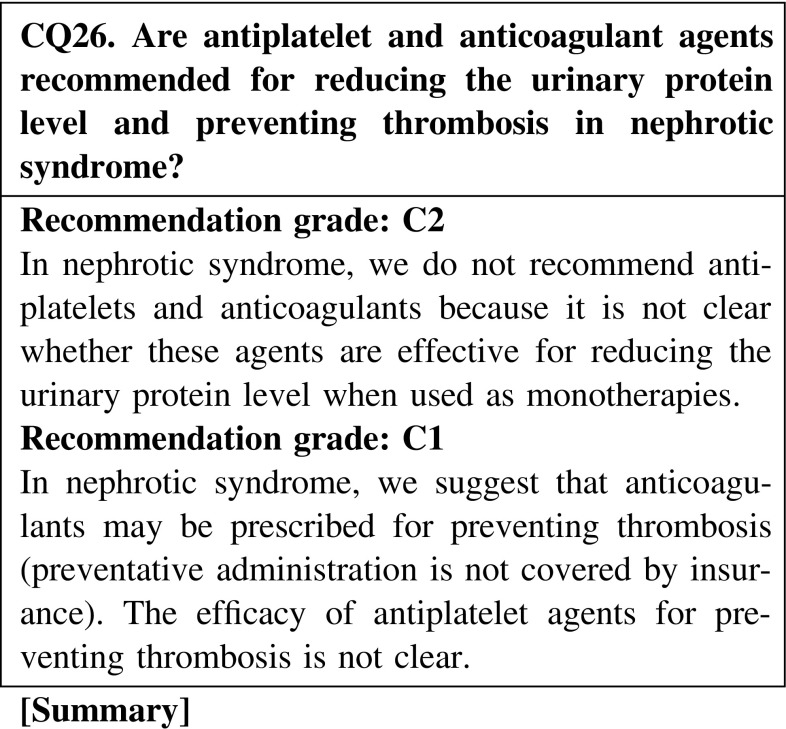


There is very little evidence to suggest that urinary protein levels are reduced in patients with nephrotic syndrome by antiplatelet and anticoagulant monotherapies; thus, their effectiveness is unclear. However, warfarin has been reported to reduce the incidence of fatal pulmonary embolism.
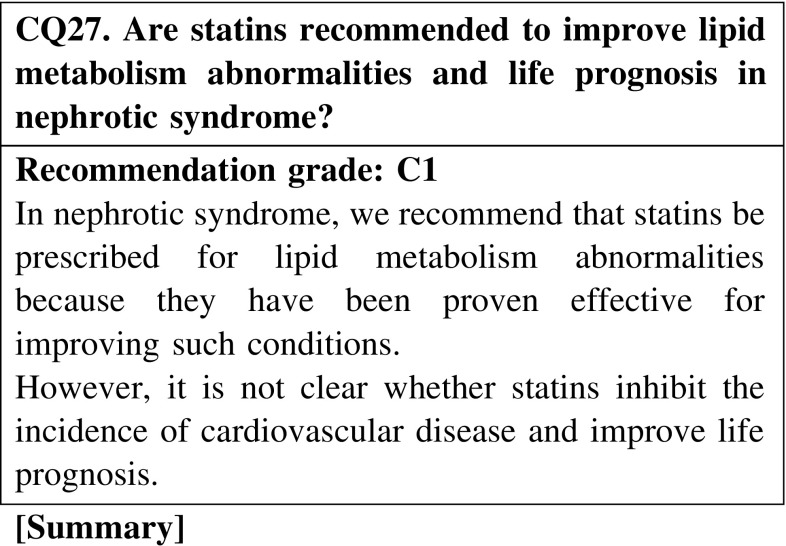


Statins can lower triglyceride, total cholesterol, and LDL cholesterol levels and increase HDL cholesterol levels in patients with nephrotic syndrome, similar to its effect in healthy persons. However, there are no prospective studies with primary endpoints such as the prevention of cardiovascular disease or the improvement of life prognosis, and its effectiveness on prognosis is unclear.
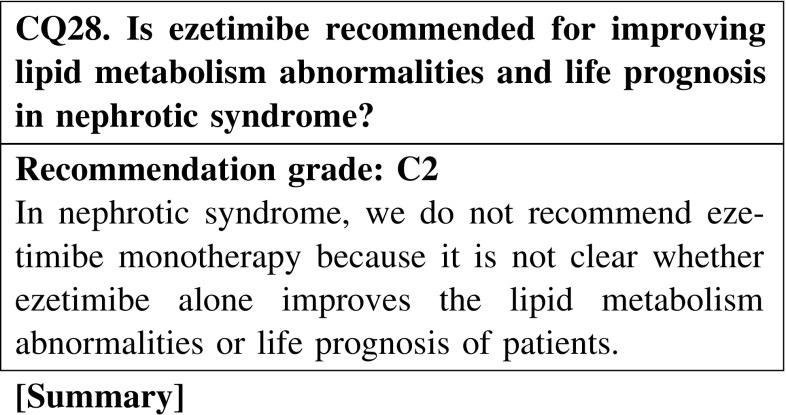


Studies verifying the clinical effect of ezetimibe monotherapy in patients with nephrotic syndrome have not been conducted, and the effect of this treatment on improving dyslipidemia or life prognosis is unclear.
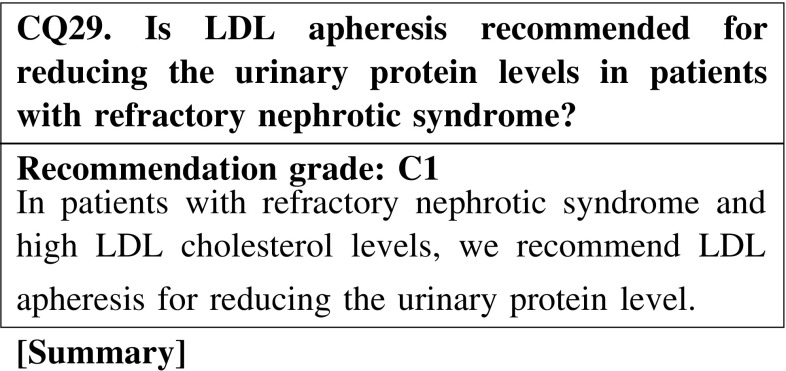


LDL apheresis is reported to be effective in reducing the urinary protein levels in approximately 50 % of cases of refractory nephrotic syndrome.
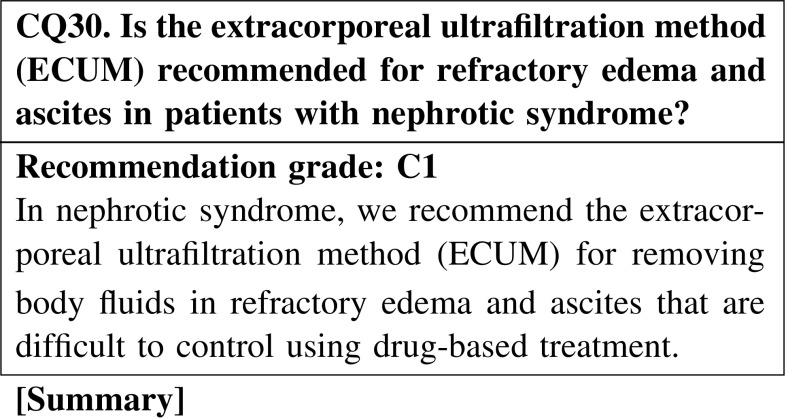


ECUM has been reported be effective in improving edema and ascites in patients with nephrotic syndrome.
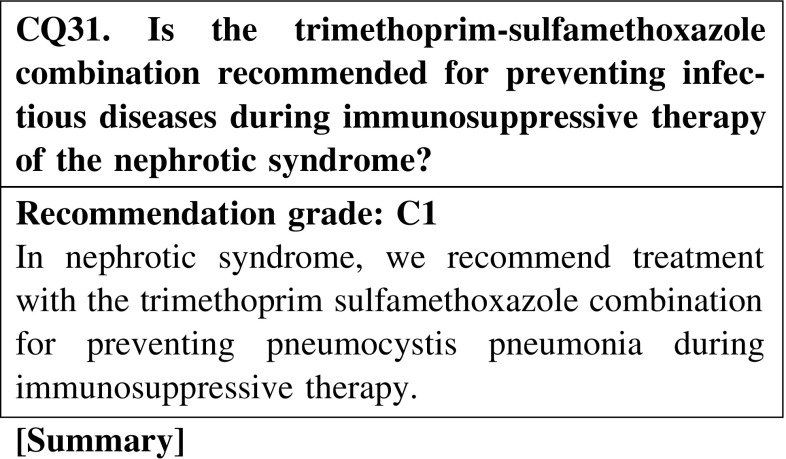


Although there are no direct evidences in nephrotic syndrome, guidelines for other similar immunosuppressive conditions recommend the prophylactic administration of the trimethoprim-sulfamethoxazole combination for pneumocystis pneumonia. Therefore, this drug combination is recommended for preventing pneumocystis pneumonia during immunosuppressive therapy of nephrotic syndrome.
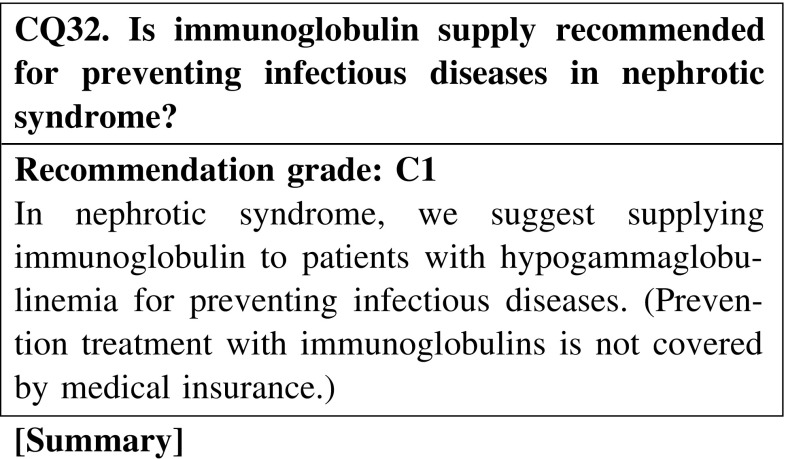


Although there is limited evidence, immunoglobulin supply could prevent infectious diseases in patients with nephrotic syndrome presenting with hypogammaglobulinemia. However, the risks and economic disadvantages of this treatment should be carefully considered.
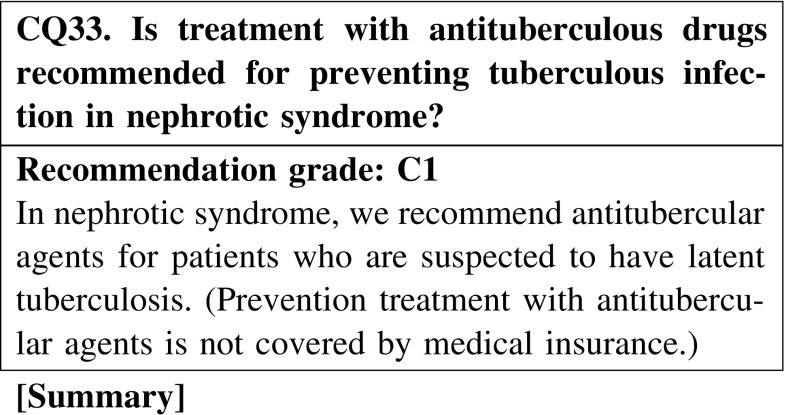


Immunosuppressive therapy for nephrotic syndrome increases the risk of progression of latent tuberculosis to active tuberculosis. There are few reports about the treatment of latent tuberculosis in patients with nephrotic syndrome; however, this treatment is necessary in patients with nephrotic syndrome undergoing immunosuppressive therapy.
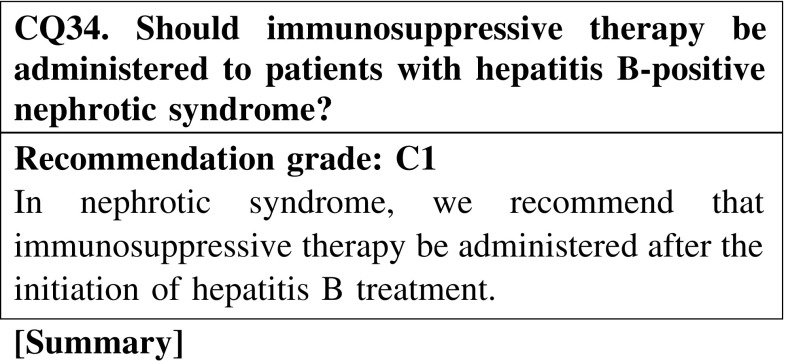


Before administering immunosuppressive therapy for nephrotic syndrome, hepatitis B infection should be evaluated first. In case infection is present, immunosuppressive therapy should be administered after the treatment of hepatitis B infection.

*8. Lifestyle and dietary instruction*
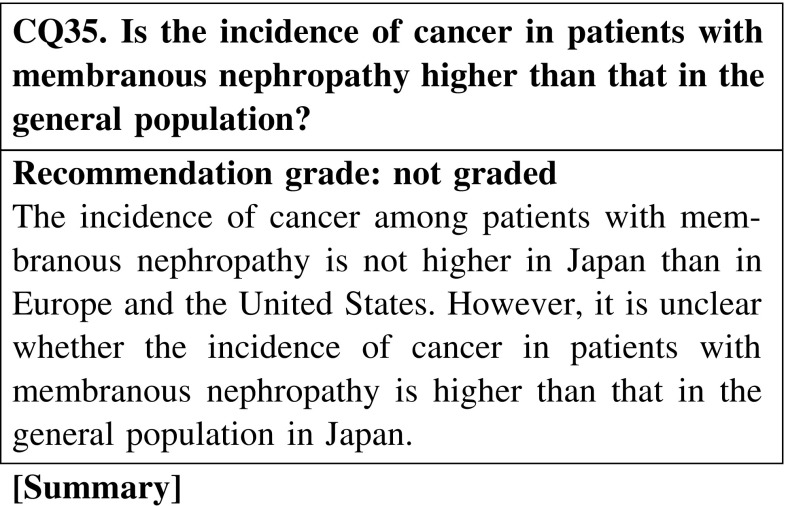


The incidence of cancer in patients with membranous nephropathy is lower n Japanese patients than in Europeans and Americans. However, it is unclear whether the incidence of cancer in patients with membranous nephropathy is higher than that in the general population in Japan.
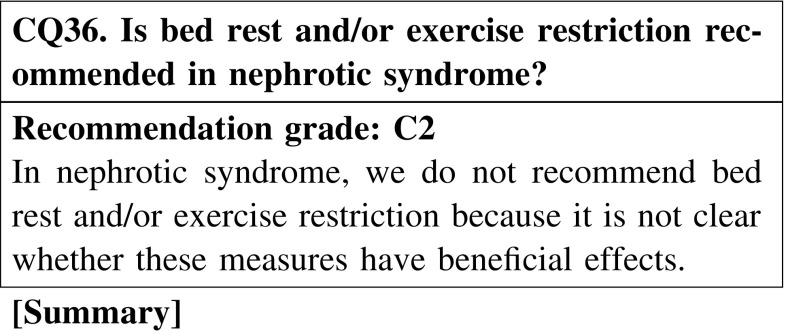


There have been no studies directly proving the beneficial effects of bed rest or exercise restriction in patients with nephrotic syndrome. Excessive bed rest is undesirable from the viewpoint of preventing pulmonary thrombosis and embolism, as well as deep vein thrombosis due to the hypercoagulable condition of nephrotic syndrome and the congestive condition associated with long-term bed rest. Moderate exercise is considered acceptable.
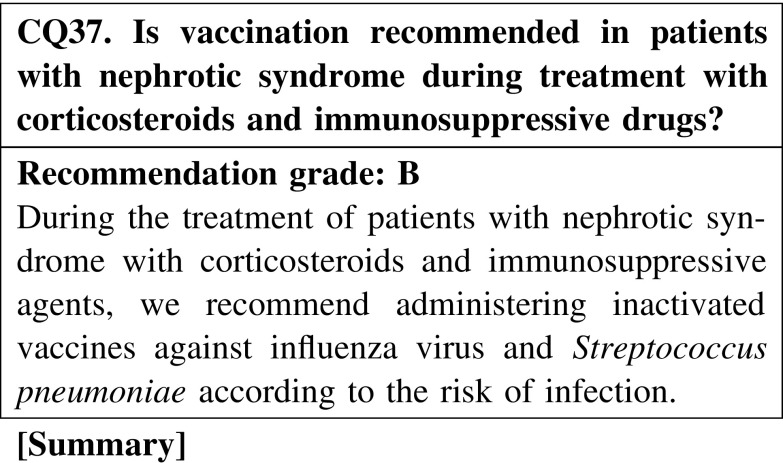


Few studies have proved the direct blocking effect of vaccination against influenza virus and *Pneumococcus* in patients with nephrotic syndrome undergoing treatment with steroid or immunosuppressive agents. Nephrotic patients have a high infection risk, and vaccination can provide safety benefits for these patients. Therefore, we recommend vaccination in patients with nephrotic syndrome, except in cases where vaccination is inappropriate. However, the efficacy and safety of live vaccine in nephrotic syndrome are controversial.
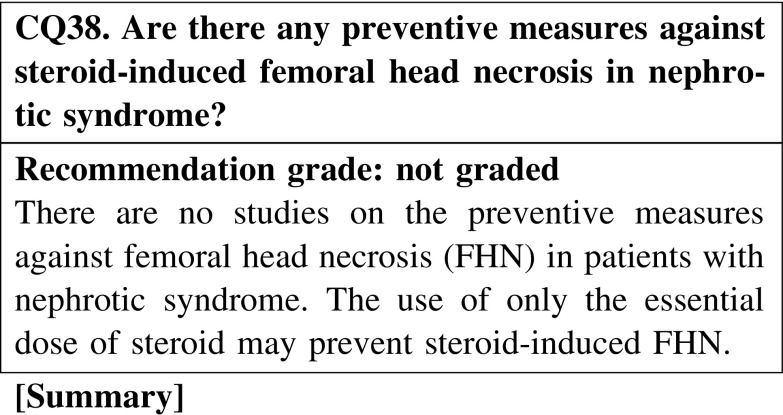


No study has directly evaluated the preventive measures for steroid-induced FHN. In nephrotic syndrome, avoiding the excess use of steroid may prevent steroid-induced FHN.
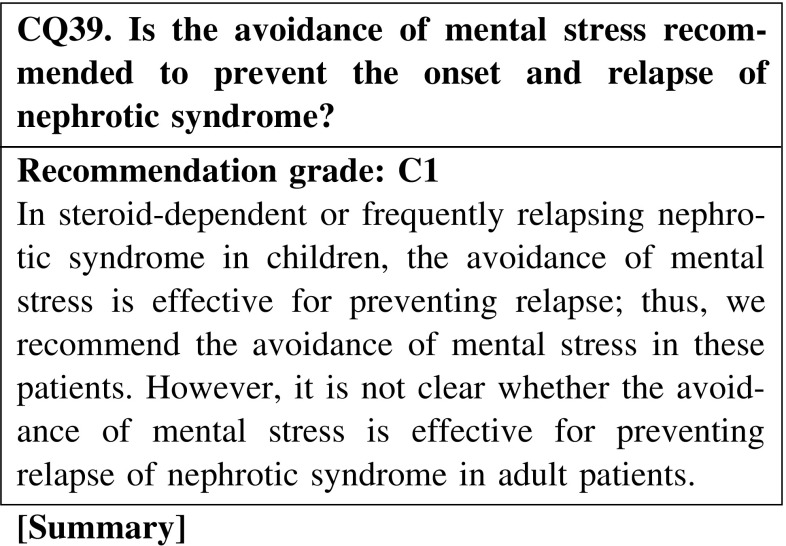


There have been no reports that evaluated the relation between the new onset of nephrotic syndrome and mental stress. In children with nephrotic syndrome, the strong relation between the relapse of nephrotic syndrome and mental stress has already been suggested. However, the relation between the onset or relapse of nephrotic syndrome and mental stress in adulthood has not been investigated thus far. Further studies are required in the future.
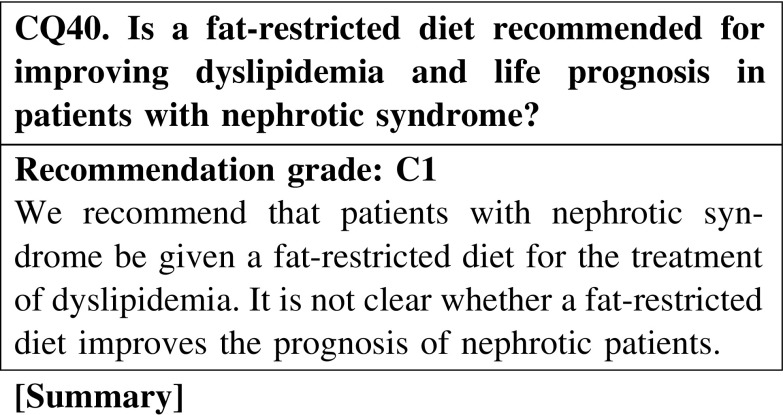


In patients with nephrotic syndrome, a fat-restricted diet consisting of low cholesterol-containing food and vegetables/beans ameliorates dyslipidemia. No study has proved that a fat-restricted diet improves the life prognosis of these patients.

### 2. Dietary Instruction

Salt restriction is essential for the alleviation of edema in nephrotic syndrome. Some patients with nephrotic syndrome show inhibited plasma renin activity (PRA) and elevated atrial natriuretic peptide (ANP) level that are comparable to the condition of salt accumulation described in the overfilling hypothesis. The efficacy of strict protein restriction is controversial; therefore, extreme protein restriction is not recommended in patients with nephrotic syndrome. The published guideline from the Japanese Society of Nephrology, “Guidelines of lifestyle and diet therapy for patients with chronic kidney disease,” recommends a protein intake of 1.0–1.1 g/kg body weight (BW)/day in minimal change nephrotic syndrome and 0.8 g/kg BW/day in other nephrotic syndromes. To keep the nitrogen balance, a calorie intake of 35 kcal/kg BW/day is recommended.

### 3. Treatment Interpretation and Treatment Algorithm

We summarized the treatments by histological types. The treatment strategies and the statements or answers of related clinical questions are comprehensively described for each strategy. Concerning adjunctive and supportive treatments or lifestyle and dietary instructions, some of the statements or answers of related clinical questions are listed.

The treatments mentioned here referred to the previous Japanese guideline “Clinical guideline for refractory nephrotic syndrome 2002” and the second revised version, “Clinical guideline for nephrotic syndrome 2011,” published by the Research Group on Progressive Renal Disease of the Ministry of Health, Labor and Welfare. Additionally a novel idea is introduced in the treatment strategies based on published papers.

Unfortunately, we could not endorse all of mentioned treatment strategies and treatment algorithms through our clinical questions. We provided the clinical questions to a maximum extent for decision making.

Patients with nephrotic syndrome are aging; thus, they have many medical complications. Treatment for these patients must be decided on a case-by-case basis rather than strictly adFigurehering to the guidelines. For the selection of agents, we provide the opinions of members of the guideline committee, with reference to the previous two guidelines in Japan. We consider that we cannot use the same types or doses of agents as those recommended by articles published overseas.

Use of treatment agents not allowed by medical insurance depends on the decision established in 2013, when the present guideline is published. In the future, this decision may change.

### 4. Minimal change nephrotic syndrome (MCNS)

#### 1. Initial treatment

Oral prednisolone is administered at a single daily dose starting at 0.8–1 mg kg^−1^ day^−1^ (maximum 60 mg/day), and continued for 1–2 weeks after remission. Thus, the initial dose is maintained for 2–4 weeks. Tapering of prednisolone is performed through the following program: a 5–10 mg dose reduction every 2–4 weeks. After the prednisolone dose is reduced to 5–10 mg/day, the minimum dose must be continued for preventing relapse for approximately 2 years, and then gradually tapered and discontinued.

**Fig. 1:**
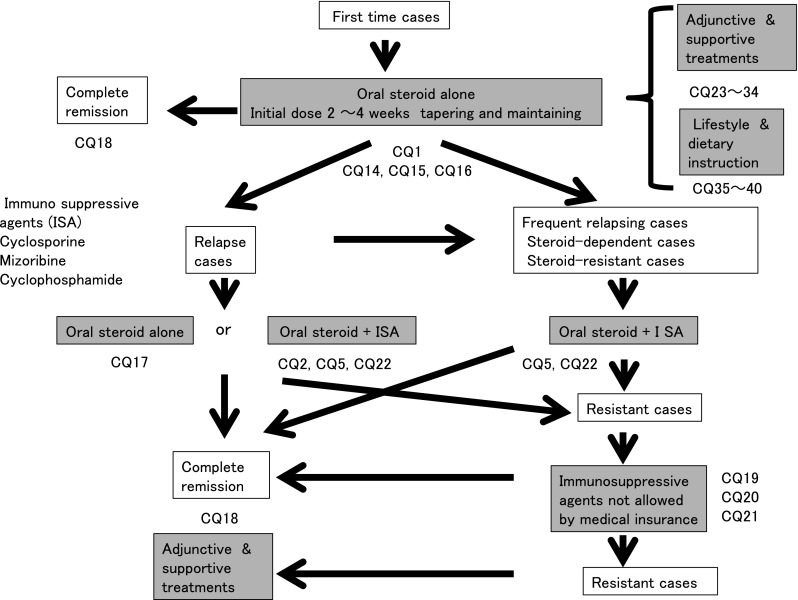
Treatment of MCNS

Steroid pulse therapy should not be performed readily; however, it may be considered for cases in which absorption of steroid from the gastrointestinal tract is doubted (Fig. 1)

- In the evaluation of efficacy, MCNS shows a high response rate to initial oral steroid treatment (CQ1).
- We recommend that oral steroid alone be prescribed for preventing the acute decline of renal function at the initial treatment (CQ1).
- Steroid pulse therapy may be considered when absorption of oral steroids seems difficult (CQ1).
- Oral steroid administration should be considered on days when patients are not receiving steroid pulse treatment (CQ14).
- In patients with severe intestinal edema associated with systemic edema, we suggest that increasing the dose of oral steroid or changing the administration routes be considered (CQ15).
- The efficacy of alternate-day steroid administration is not clear because there are few relevant reports in adult nephrotic syndrome (CQ16).
- There is no clear goal about the duration of continued steroid therapy after remission; however, at least 24 weeks may be necessary in MCNS (CQ18).

#### 2. Relapse cases

Steroids are administered at equal or lower doses than the initial dose at the relapse of nephrotic syndrome.

- As for steroid therapy for recurrent nephrotic syndrome, the opinions differ as to whether the dose of treatment should the same as that of the first treatment or lower than that of the first treatment (CQ17).

#### 3. Frequently relapsing, steroid-dependent, and steroid-resistant cases

Immunosuppressive agents such as cyclosporine (1.5–3.0 mg kg^−1^ day^−1^), cyclophosphamide (50–100 mg/day), or mizoribine (150 mg/day) are administered in addition to steroid.

The efficacy of mizoribine has been confirmed in children but not in adults. Therefore, the choice of mizoribine for adult patients is suggested here.

During treatment with immunosuppressive agents, the patient’s age and complications should be considered. Elderly patients easily develop complications.

- In MCNS, we recommend that cyclosporine with steroid be prescribed for reducing the urinary protein level in steroid-resistant and relapse cases (CQ2, CQ5).
- In frequently relapsing nephrotic syndrome derived from MCNS and FSGS in adult patients, we recommend cyclosporine or cyclophosphamide be additionally prescribed to steroid for reducing the urinary protein level (CQ5).
- The addition of mizoribine to steroid decreases the relapse rate in children with frequently relapsing nephrotic syndrome, whereas it is not known whether the same is true in adults. Mizoribine may be considered depending on the cases (CQ5).
- In steroid-dependent or steroid-resistant nephrotic syndrome derived from MCNS and FSGS, we recommend cyclosporine or cyclophosphamide be additionally administered with steroid for reducing the urinary protein level (CQ5).
- Recently, MCNS is found even in elderly patients. Few clinical studies have evaluated the efficacy of immunosuppressive agents in elderly patients with nephrotic syndrome; however, the efficacy of these agents for reducing the urine protein level was reported to be similar to that in younger patients. The incidence rate of adverse effects in elderly patients is higher than that in younger patients. Careful observation is necessary in the treatment of elderly patients with nephrotic syndrome (CQ22).

#### 4. Immunosuppressive agents not covered by medical insurance (at the time of description of this guideline in 2013)

The use of agents not covered by medical insurance in Japan, such as rituximab, mycophenolate mofetil, and azathioprine, may be considered for patients resistant to agents allowed by medical insurance. However, it is not clear whether these agents are effective for reducing the urinary protein level and preventing the decline of renal function in nephrotic syndrome. For patients with frequently relapsing or steroid-resistant nephrotic syndrome, we suggest that these agents may be effective for reducing the urinary protein level and preventing the decline of renal function (CQ19, CQ20, CQ21).

### 5. Focal segmental glomerulosclerosis (FSGS)

#### 1. Initial treatment

**Fig. 2:**
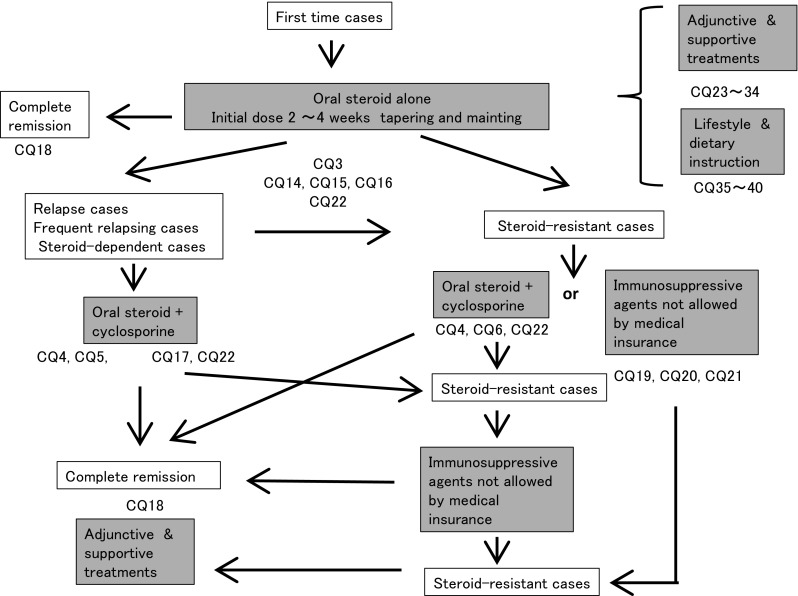
Treatment of FSGS

Oral prednisolone is administered at a single daily dose starting at 0.8–1 mg kg^−1^ day^−1^ (maximum 60 mg/day) for 2–4 weeks as the initial treatment. Steroid pulse therapy is considered for cases with massive urine protein excretion or severe systemic edema. After remission, tapering of steroid dose is performed following the program of MCNS (Fig. 2).

- Oral steroid therapy as an initial treatment is effective for FSGS, showing a remission induction rate from 20 to 50 %. We recommend steroid therapy as the initial treatment (CQ3).
- Steroid pulse therapy may be considered for patients with severe intestinal edema (CQ3).
- Oral steroid should be administered on days when patients are not receiving steroid pulse treatment (CQ14).
- In patients with severe intestinal edema associated with systemic edema, we suggest increasing the oral steroid or changing the prescription routes (CQ15).
- The efficacy of alternate-day steroid administration is not clear in preventing the adverse effects of steroid (CQ16).
- There is no clear goal about the duration of continued steroid use after remission; however, steroid was continually used for at least 6 months in observational studies in patients with FSGS (CQ18).
- The efficacy of immunosuppressive agents for reducing the urine protein level in elderly patients was reported to be similar to that in younger patients. The incidence rate of adverse effects in elderly patients is higher than that in younger patients. Careful observation is necessary in the treatment of elderly patients with nephrotic syndrome. The selection of steroid treatment or combination treatment with steroid and immunosuppressive agents should be determined on the basis of the age or complications of patients (CQ22).

#### 2. Relapsing and frequently relapsing cases

The combination of oral steroid and cyclosporine, 2.0–3.0 mg kg^−1^ day^−1^, is selected for relapsing and frequently relapsing cases.

- The combination of oral steroid and cyclosporine is selected for patients with relapsing and frequently relapsing FSGS instead of steroid-alone treatment (CQ5, CQ17, CQ22).

#### 3. Steroid-dependent and steroid-resistant cases

If steroid-alone treatment for >4 weeks fails to attain complete or incomplete remission, cyclosporine, 2.0–3.0 mg kg^−1^ day^−1^, is added to steroid therapy.

- Compared with steroid-alone treatment, the combination treatment of cyclosporine and steroid may be more effective for reducing the urinary protein level in steroid-resistant FSGS. The nephrotoxicity of cyclosporine due to long-term use is unclear (CQ4).
- It is not clear whether cyclosporine is more effective than mizoribine or cyclophosphamide for reducing the urinary protein level (CQ6).
- The efficacy of immunosuppressive agents for reducing the urine protein level in elderly patients was reported to be similar to that in younger patients. The incidence rate of adverse effects in elderly patients is higher than that in younger patients. Careful observation is necessary in the treatment of elderly patients with nephrotic syndrome. The selection of steroid treatment or combination treatment with steroid and immunosuppressive agents should be determined on the basis of the age or complications of patients (CQ22).

#### 4. Immunosuppressive agents not covered by medical insurance (at the time of description of this guideline in 2013)

The use of agents not covered by medical insurance in Japan, such as rituximab, mycophenolate mofetil, and azathioprine, may be considered for patients resistant to agents covered by medical insurance. However, it is not clear whether these agents are effective for reducing the urinary protein level and preventing the decline of renal function in nephrotic syndrome. In cases of frequently relapsing or steroid-resistant nephrotic syndrome, we suggest that these agents may be effective for reducing the urinary protein level and preventing the decline of renal function (CQ19, CQ20, CQ21).

### 6. Membranous nephropathy (MN)

#### 1. Initial treatment

**Fig. 3:**
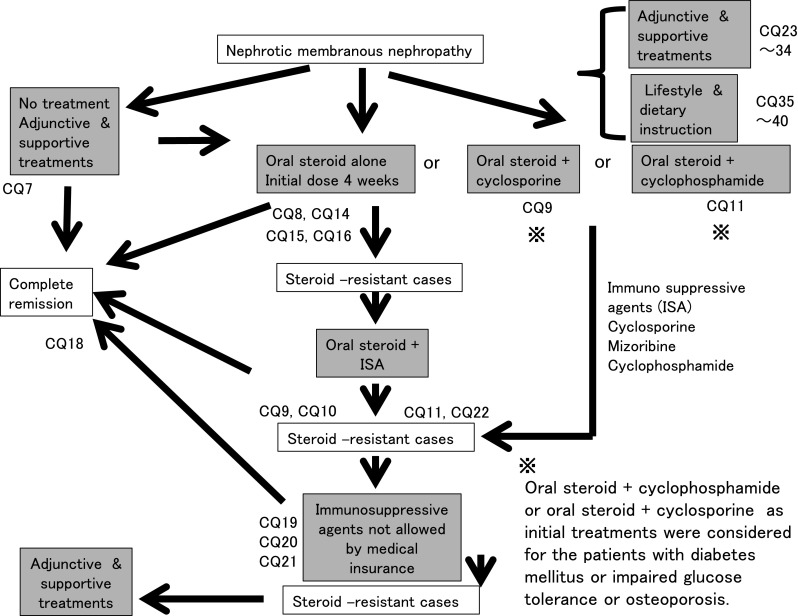
Treatment of membranous nephropathy

Oral prednisolone is administered at a single daily dose starting at 0.6–0.8 mg kg^−1^ day^−1^ and continued for 4 weeks. Instead of oral steroid alone, prednisolone and cyclophosphamide are administered as a starting dose of 50–100 mg/day. Lower-dose oral steroid and cyclosporine as the initial treatment is considered for patients who are concerned about the adverse effects of steroids, such as diabetic patients (Fig. 3).

- In some patients with MN with nephrotic syndrome, we suggest that no treatment or supportive treatment alone without immunosuppressive agents may reduce the urinary protein level. However, we cannot expect that no treatment or supportive treatment alone is effective for preventing the decline of renal function (CQ7).
- Steroid-alone treatment is not more effective than no treatment for reducing the urinary protein level. We recommend steroid-alone treatment for preventing the decline of renal function (CQ8).
- In a retrospective study on Japanese patients with MN, the remission rates did not show any significant differences between three treatment groups (steroid alone, steroid and cyclophosphamide, and supportive treatment); however, treatment with steroid alone and the combination of steroid and cyclophosphamide showed significant effectiveness in preventing the decline of renal function when compared with supportive treatment (CQ8).
- In steroid-resistant MN, we recommend the combination of steroid and cyclosporine for reducing the urinary protein level and preventing the decline of renal function (CQ9).
- Between steroid with cyclosporine and steroid with alkylating agents, the superiority of the treatment with steroid with cyclosporine has not been recognized (CQ9).
- In patients with severe intestinal edema associated with systemic edema, we suggest increasing the dose of oral steroid or changing the prescription (CQ15).
- The efficacy of alternate-day administration is not clear in preventing the adverse effects of steroid (CQ16).
- There is no clear goal about the period of continued steroid administration after remission; however, steroid was continued for at least 6 months in observational studies on patients with MN (CQ18).
- The efficacy of immunosuppressive agents for reducing the urine protein level in elderly patients was reported to be similar to that in younger patients. The incidence rate of adverse effects in elderly patients is higher than that in younger patients. Careful observation is necessary in the treatment of elderly patients with nephrotic syndrome. The selection of steroid treatment or combination treatment with steroid and immunosuppressive agents should be determined on the basis of the age or complications of patients (CQ22).

#### 2. Steroid-resistant cases

If steroid-alone treatment for >4 weeks fails to attain complete or incomplete remission, cyclosporine (2.0–3.0 mg/kg/day), mizoribine (150 mg/day), or cyclophosphamide (50–100 mg/day) is added to steroid therapy.

- In steroid-resistant MN, we recommend the combination of steroid and cyclosporine for reducing the urinary protein level and preventing the decline of renal function (CQ9).
- In steroid-resistant or refractory MN, we suggest that the addition of mizoribine to steroid is effective for reducing the urinary protein level (CQ10).
- In MN, we recommend the addition of cyclophosphamide to steroid for reducing the urinary protein level and preventing the decline of renal function (CQ11). Because of the frequent adverse effects of alkylating agents and the limited evidence of the efficacy of these agents in Japanese patients, we suggest that the use of alkylating agents be considered carefully.

#### 3. Non-nephrotic cases

- In patients with MN showing non-nephrotic proteinuria, we suggest that conservative treatment with RAS inhibitors, lipid-lowering agents, or antiplatelet agents is effective for reducing the urinary protein level in some cases. (CQ12).
- However, it is not clear whether those conservative treatments are effective for preventing the decline of renal function (CQ12).

### 7. Membranoproliferative glomerulonephritis (MPGN)

- In children with MPGN, steroid is recommended for reducing the urinary protein level and preventing the decline of renal function. In adult cases, the efficacy of steroid is unclear, although steroid may be considered in some patients with MPGN (CQ13).

### 8. Adjunctive and supportive treatments

#### 1. Renin-angiotensin system (RAS) inhibitors

- In patients with hypertension and nephrotic syndrome, we recommend RAS inhibitors be prescribed for reducing the urinary protein level. It is not known whether RAS inhibitors are effective for patients with nephrotic syndrome without hypertension (CQ23).

#### 2. Diuretics

- In edematous patients with nephrotic syndrome, we recommend oral diuretics, particularly loop diuretics, be prescribed for reducing edema. The use of intravenous diuretics should be considered if the effect of oral diuretics is insufficient, because they effectively reduce body fluid volumes (CQ24).

#### 3. Albumin agents

- Albumin administration does not improve hypoalbuminemia or edema in patients with nephrotic syndrome and may exacerbate hypertension; therefore, it is not recommended for this condition. However, in cases of severe shock or pulmonary edema, albumin administration may have a temporary but useful effect (CQ25).

#### 4. Antiplatelet and anticoagulant agents

- For patients with nephrotic syndrome, we do not recommend prescribing antiplatelets and anticoagulants as monotherapies because their effectiveness in reducing the urinary protein level is not clear. We suggest that administration of anticoagulants may be prescribed for preventing thrombosis (preventative administration is not covered by insurance). The efficacy of antiplatelet agents for preventing thrombosis is not clear (CQ26).

#### 5. Statins

- In nephrotic syndrome, we recommend statins be prescribed for lipid metabolism abnormalities because they have been proven effective for improving such conditions. However, it is not clear whether statins reduce the incidence of cardiovascular disease and improve prognosis (CQ27).

#### 6. Ezetimibe

- In nephrotic syndrome, it is not clear whether this treatment improves the lipid metabolism abnormalities or prognosis of patients (CQ28).

#### 7. Low-density lipoprotein (LDL) apheresis

- In patients with refractory nephrotic syndrome and high LDL cholesterol levels, we recommend LDL apheresis for reducing the urinary protein level (CQ29).

#### 8. Extracorporeal ultrafiltration method (ECUM)

- In patients with nephrotic syndrome, we recommend the ECUM for the removal of body fluids in refractory edema and ascites that are difficult to control using drug-based therapy (CQ30).

#### 9. Trimethoprim-sulfamethoxazole combination

- In patients with nephrotic syndrome, we recommend treatment with the trimethoprim-sulfamethoxazole combination for preventing pneumocystis pneumonia during immunosuppressive therapy (CQ31).

#### 10. Immunoglobulin supply

- In nephrotic syndrome, we suggest supplying immunoglobulin to patients with hypogammaglobulinemia for the prevention of infectious diseases. (Prevention treatment with immunoglobulin supply is not covered by medical insurance.) (CQ32).

#### 11. Antituberculous drugs

- We recommend antitubercular agents be given for patients with nephrotic syndrome who are suspected to have latent tuberculosis. (Prevention treatment with antitubercular agents is not covered by medical insurance.) (CQ33)

#### 12. Hepatitis B virus treatment

- In patients with nephrotic syndrome, we recommend that immunosuppressive therapy be started after the initiation of hepatitis B treatment (CQ34).

### 9. Lifestyle and dietary instruction

#### 1. Screening for cancer

- The incidence of cancer in patients with membranous nephropathy is not higher in Japan than in Europe and the United States. However, it is unclear whether the incidence of cancer in patients with membranous nephropathy is higher than that in the general population in Japan (CQ35).

#### 2. Bed rest and/or exercise restriction

- We do not recommend bed rest and/or exercise restriction for patients with nephrotic syndrome because it is not clear whether these measures have beneficial effects (CQ36).

#### 3. Vaccination

- During the treatment with corticosteroids and immunosuppressive agents, we recommend administering inactivated vaccines against influenza virus and *Streptococcus pneumoniae* according to the risk of infection to patients with nephrotic syndrome (CQ37).

#### 4. Steroid-induced femoral head necrosis (FHN)

- No study has investigated the preventive measures against FHN in patients with nephrotic syndrome. The use of only the essential dose of steroid may prevent the development of steroid-induced FHN (CQ38).

#### 5. Avoidance of mental stress

- In steroid-dependent and/or frequently relapsing nephrotic syndrome in children, avoidance of mental stress is effective to prevent relapse; thus, we recommend the avoidance of mental stress in these patients. However, it is not clear whether avoidance of mental stress is effective for preventing the relapse of nephrotic syndrome in adults (CQ39).

#### 6. Fat-restricted diet

- We recommend providing fat-restricted diet for the treatment of dyslipidemia in patients with nephrotic syndrome. It is not clear whether a fat-restricted diet improves the prognosis of nephrotic patients (CQ40).

##### **Acknowledgments**

All authors are Advisory Committee of Clinical Guidelines for Nephrotic Syndrome 2014. Committee chairman: Shinichi Nishi. Committee members: Yoshifumi Ubara, Yasunori Utsunomiya, Koichi Okada, Yoko Obata, Hiroyasu Kai, Hideyasu Kiyomoto, Shin Goto, Tsuneo Konta, Yoshie Sasatomi, Yoshinobu Sato, Tomoya Nishino, Kazuhiko Tsuruya, Kengo Furuichi, Junichi Hoshino, Yasuhiro Watanabe. Chief Chairman of the Clinical Practice Guidelines for Progressive Kidney Diseases: Kenjiro Kimura. Leader of the Research for Progressive Kidney Diseases of the Ministry of Health, Labour and Welfare: Seiichi Matsuo. Cooperative Medical Society: The Japanese Association for Infectious Diseases, The Japanese Society for Pediatric Nephrology, The Japanese Society of Nephrology.

**Bibliography**

I. Disease entity · definition (pathogenesis)
  1. Russo LM, et al. The normal kidney filters nephrotic levels of albumin retrieved by proximal tubule cells:retrieval is disrupted in nephrotic states. Kidney Int 2007;71:504–13.

II. Diagnosis

1. Symptomatology · clinical manifestation
  1. Abdel–Hafez M, et al. Idiopathic nephrotic syndrome and atopy:Is there a common link? Am J Kidney Dis 2009;54:945–53.
  2. Yokoyama H, et al.;On the behalf of the Committee for the Standardization of Renal Pathological Diagnosis and for Renal Biopsy and Disease Registry in the Japanese Society of Nephrology. Membranous nephropathy in Japan:analysis of the Japan Renal Biopsy Registry(J-RBR). Clin Exp Nephrol 2012;16:557–63.
  3. Andoh D, et al. Loss of nocturnal decline of blood pressure in non-diabetic patients with nephrotic syndrome in the early and middle stages of chronic kidney disease. Hypertens Res 2009;32:364–8.
  4. Mahmoodi BK, et al. High absolute risks and predictors of venous and arterial thromboembolic events in patients with nephrotic syndrome:results from a large retrospective cohort study. Circulation 2008;117:224–30.
  5. Witz M, et al. Renal vein occlusion:diagnosis and treatment. Isr Med Assoc J 9:402–5, 2007.
  6. Glassock RJ. Attending rounds:an older patient with nephrotic syndrome. Clin J Am Soc Nephrol 2012;7:665–70.

2. Laboratory findings
  1. Ginsberg JM, et al. Use of single voided urine samples to estimate quantitative proteinuria. N Engl J Med 1983;309:1543–6.
  2. Bazzi C, et al. A modern approach to selectivity of proteinuria and tubulointerstitial damage in nephrotic syndrome.Kidney Int 2000;58:1732–41.
  3. Vaziri ND:Molecular mechanisms of lipid disorders in nephrotic syndrome. Kidney Int 2003;63:1964–76.
  4. Joven J, et al. Abnormalities of lipoprotein metabolism in patients with the nephrotic syndrome. N Engl J Med 1990;323:579–84.
  5. Kerlin BA, et al. Epidemiology and pathophysiology of nephrotic syndrome-associated thromboembolic disease. Clin J Am Soc Nephrol 2012;7:513–20.
  6. Rabelink TJ, et al. Thrombosis and hemostasis in renal disease. Kidney Int 1994;46:287–96.
  7. Singhal R, et al. Thromboembolic complications in the nephrotic syndrome:pathophysiology and clinical management. Thromb Res 2006;118:397–407.
  8. Mahmoodi BK, et al. High absolute risks and predictors of venous and arterial thromboembolic events in patients with nephrotic syndrome:results from a large retrospective cohort study. Circulation 2008;117:224–30.
  9. Bates SM, et al. Antithrombotic Therapy and Prevention of Thrombosis, 9th ed:American College of Chest Physicians Evidence-Based Clinical Practice Guidelines. Chest 2012;141:e351S–418S.
  10. Wells PS, et al. Value of assessment of pretest probability of deep-vein thrombosis in clinical management. Lancet 1997;350:1795–8.
  11. Ogi M, et al. Risk factors for infection and immunoglobulin replacement therapy in adult nephrotic syndrome. Am J Kidney Dis 1994;24:427–36.

III. Epidemiology · prognosis
  1. Incidence · prevalence · recurrence rate
    1. Fujimoto S, et al. Minimal change nephrotic syndrome in adults:response to corticosteroid therapy and frequency of relapse. Am J Kidney Dis 1991;17:687–92.
    2. Nakayama M, et al. Steroid responsiveness and frequency of relapse in adult-onset minimal change nephrotic syndrome. Am J Kidney Dis 2002;39:503–12.
    3. Tse KC, et al. Idiopathic minimal change nephrotic syndrome in older adults:steroid responsiveness and pattern of relapses. Nephrol Dial Transplant 2003;18:1316–20.
    4. Imbasciati E, et al. Controlled trial of methylprednisolone pulses and low dose oral prednisone for the minimal change nephrotic syndrome. Br Med J(Clin Res Ed)1985;291:1305–8.
    5. Takei T, et al. The characteristics of relapse in adult-onset minimal-change nephrotic syndrome. Clin Exp Nephrol 2007;11:214–7.
    6. Matsumoto H, et al. Favorable outcome of low-dose cyclosporine after pulse methylprednisolone in Japanese adult minimal-change nephrotic syndrome. Intern Med 2004;43:668–73.
    7. Lee HY, et al. The efficacy of cyclosporine A in adult nephrotic syndrome with minimal change disease and focal-segmental glomerulosclerosis: a multicenter study in Korea. Clin Nephrol 1995;43:375–81.
    8. Shibasaki T, et al. A randomized open-label comparative study of conventional therapy versus mizoribine onlay therapy in patients with steroid-resistant nephrotic syndrome (postmarketing survey). Clin Exp Nephrol 2004;8:117–26.
    9. Fujinaga S, et al. Single daily high-dose mizoribine therapy for children with steroid-dependent nephrotic syndrome prior to cyclosporine administration. Pediatr Nephrol 2011;26:479–83.
    10. Muso E, et al. Low density lipoprotein apheresis therapy for steroid-resistant nephrotic syndrome. Kansai-FGS-Apheresis Treatment (K-FLAT)Study Group. Kidney Int Suppl 1999;71:S122–5.
    11. Matalon A, et al. Treatment of focal segmental glomerulosclerosis. Semin Nephrol 2000;20:309–17.
    12. Korbet SM. Treatment of primary focal segmental glomerulosclerosis. Kidney Int 2002;62:2301–10.
    13. Kirpekar R, et al. Clinicopathologic correlates predict the outcome in children with steroid-resistant idiopathic nephrotic syndrome treated with pulse methylprednisolone therapy. Am J Kidney Dis 2002;39:1143–52.
    14. Chang JW, et al. Low-dose methylprednisolone pulse therapy in Chinese children with steroid resistant focal segmental glomerulosclerosis. Pediatr Int 2007;49:349–54.
    15. Pena A, et al. Steroid-resistant nephrotic syndrome:long-term evolution after sequential therapy. Pediatr Nephrol 2007;22:1875–80.
    16. Meyrier AY. Treatment of focal segmental glomerulosclerosis with immunophilin modulation:when did we stop thinking about pathogenesis? Kidney Int 2009;76:487–91.
    17. Meyrier A. An update on the treatment options for focal segmental glomerulosclerosis. Expert Opin Pharmacother 2009;10:615–28.
    18. Tojo K, et al. Possible therapeutic application of low density lipoprotein apheresis (LDL-A) in conjunction with double filtration plasmapheresis (DFPP) in drug-resistant nephrotic syndrome due to focal glomerular sclerosis (FGS). Jpn J Nephrol 1988;30:1153–60.
    19. Hattori M, et al. A combined low-density lipoprotein apheresis and prednisone therapy for steroid-resistant primary focal segmental glomerulosclerosis in children. Am J Kidney Dis 2003;42:1121–30.
    20. Muso E, et al. Beneficial effect of low-density lipoprotein apheresis (LDL-A) on refractory nephrotic syndrome (NS) due to focal glomerulosclerosis (FGS). Clin Nephrol 2007;67:341–4.
    21. Shiiki H, et al. Prognosis and risk factors for idiopathic membranous nephropathy with nephrotic syndrome in Japan. Kidney Int 2004;65:1400–7.
    22. Kida H, et al. Long - term prognosis of membranous nephropathy. Clin Nephrol 1986;25:64–9.
    23. Troyanov S, et al. Idiopathic membranous nephropathy:definition and relevance of a partial remission. Kidney Int 2004;66:1199–205.
    24. Bazzi C, et al. A modern approach to selectivity of proteinuria and tubulointerstitial damage in nephrotic syndrome. Kidney Int 2000;58:1732–41.
    25. Schieppati A, et al. Prognosis of untreated patients with idiopathic membranous nephropathy. N Engl J Med 1993;329:85–9.
    26. Cattran DC, et al. Cyclosporin in idiopathic glomerular disease associated with the nephrotic syndrome:workshop recommendations. Kidney Int 2007;72:1429–47.
    27. Neary JJ, et al. Linkage of a gene causing familial membranoproliferative glomerulonephritis type III to chromosome 1. J Am Soc Nephrol 2002;13:2052–7.
    28. Smith RJ, et al. New approaches to the treatment of dense deposit disease. J Am Soc Nephrol 2007;18:2447–56.
    29. Little MA, et al. Severity of primary MPGN, rather than MPGN type, determines renal survival and post-transplantation recurrence risk. Kidney Int 2006;69:504–11.
    30. Cameron JS, et al. Idiopathic mesangiocapillary glomerulonephritis. Comparison of types and in children and adults and long-term prognosis. Am J Med 1983;74:175–92.
    31. Zäuner I, et al. Effect of aspirin and dipyridamole on proteinuria in idiopathic membranoproliferative glomerulonephritis:a multicentre prospective clinical trial. Collaborative Glomerulonephritis Therapy Study Group (CGTS). Nephrol Dial Transplant 1994;9:619–22.
    32. Jones G, et al. Treatment of idiopathic membranoproliferative glomerulonephritis with mycophenolate mofetil and steroids. Nephrol Dial Transplant 2004;19:3160–4.
    33. Waldman M, et al. Adult minimal-change disease: clinical characteristics, treatment, and outcomes. Clin J Am Soc Nephrol 2007;2:445–53.
    34. Mak SK, et al. Long-term outcome of adult-onset minimal-change nephropathy. Nephrol Dial Transplant 1996;11:2192–201.
    35. Bohle A, et al. The long-term prognosis of the primary glomerulonephritides. A morphological and clinical analysis of 1747 cases. Pathol Res Pract 1992;188:908–24.
    36. Takeda Y, et al. Two cases of nephrotic syndrome (NS)-induced acute kidney injury(AKI)associated with renal hypouricemia. Clin Nephrol 2011;76:78–82.
    37. Chen CL, et al. Increased endothelin 1 expression in adult-onset minimal change nephropathy with acute renal failure. Am J Kidney Dis 2005;45:818–25.
    38. Ehrich JH, et al. Long versus standard prednisone therapy for initial treatment of idiopathic nephrotic syndrome in children. Arbeitsgemeinschaft für Pädiatrische Nephrologie. Eur J Pediatr 1993;152:357–61.
    39. Thomas DB, et al. Clinical and pathologic characteristics of focal segmental glomerulosclerosis pathologic variants. Kidney Int 2006;69:920–6.

2. Remission rate · nonresponsive rate · renal prognosis
  1. Yokoyama H, et al.;the Committee for the Standardization of Renal Pathological Diagnosis and for Renal Biopsy and Disease Registry in the Japanese Society of Nephrology. Membranous nephropathy in Japan:analysis of the Japan Renal Biopsy Registry(J-RBR). Clin Exp Nephrol 2012;16:557–63.
  2. Fujimoto S, et al. Minimal change nephrotic syndrome in adults:response to corticosteroid therapy and frequency of relapse. Am J Kidney Dis 1991;17:687–92.
  3. Yokoyama H, et al.;the Committee for the Standardization of Renal Pathological Diagnosis and for Renal Biopsy and Disease Registry of the Japanese Society of Nephrology, and the Progressive Renal Disease Research of the Ministry of Health, Labour and Welfare of Japan. Renal disease in the elderly and the very elderly Japanese:analysis of the Japan Renal Biopsy Registry(J-RBR). Clin Exp Nephrol 2012;16:903–20.
  4. Takei T, et al. The characteristics of relapse in adult-onset minimal-change nephrotic syndrome. Clin Exp Nephrol 2007;11:214–7.
  5. Nakayama M, et al. Steroid responsiveness and frequency of relapse in adult–onset minimal change nephrotic syndrome. Am J Kidney Dis 2002;39:503–12.
  6. Ponticelli C, et al. Posttransplant recurrence of primary glomerulonephritis. Clin J Am Soc Nephrol 2010;5:2363–72.
  7. Cattran DC, et al. A randomized trial of cyclosporine in patients with steroid-resistant focal segmental glomerulosclerosis. North America Nephrotic Syndrome Study Group. Kidney Int 1999;56:2220–6.
  8. Waldman M, et al. Controversies in the treatment of idiopathic membranous nephropathy. Nat Rev Nephrol 2009;5:469–79.
  9. Kalliakmani P, et al. Benefit and cost from the long-term use of cyclosporine-A in idiopathic membranous nephropathy. Nephrology(Carlton) 2010;15:762–7.

3. Incidence of complication
  1. Ordoñez JD, et al. The increased risk of coronary heart disease associated with nephrotic syndrome. Kidney Int 1993;44:638–42.
  2. Lechner BL, et al. The risk of cardiovascular disease in adults who have had childhood nephrotic syndrome. Pediatr Nephrol 2004;19:744–8.
  3. Ogi M, et al. Risk factors for infection and immunoglobulin replacement therapy in adult nephrotic syndrome. Am J Kidney Dis 1994;24:427–36.
  4. Wu HM, et al. Interventions for preventing infection in nephrotic syndrome. Cochrane Database Syst Rev 2012;4:CD003964.
  5. Uncu N, et al. Primary peritonitis in children with nephrotic syndrome:results of a 5-year multicenter study. Eur J Pediatr 2010;169:73–6.
  6. Citak A, et al. Hemostatic problems and thromboembolic complications in nephrotic children. Pediatr Nephrol 2000;14:138–42.
  7. Kayali F, et al. Venous thromboembolism in patients hospitalized with nephrotic syndrome. Am J Med 2008;121:226–30.
  8. Mahmoodi BK, et al. High absolute risks and predictors of venous and arterial thromboembolic events in patients with nephrotic syndrome:results from a large retrospective cohort study. Circulation 2008;117:224–30.
  9. Lionaki S, et al. Venous thromboembolism in patients with membranous nephropathy. Clin J Am Soc Nephrol 2012;7:43–51.
  10. Kerlin BA, et al. Epidemiology and risk factors for thromboembolic complications of childhood nephrotic syndrome:a Midwest Pediatric Nephrology Consortium (MWPNC) study. J Pediatr 2009;155:105–10.
  11. Kerlin BA, et al. Epidemiology and pathophysiology of nephrotic syndrome-associated thromboembolic disease. Clin J Am Soc Nephrol 2009;7:513–20.
  12. 12 Singhal R, et al. Thromboembolic complications in the nephrotic syndrome:pathophysiology and clinical management. Thromb Res 2006;118:397–407.
  13. Cherng SC, et al. The role of lung scintigraphy in the diagnosis of nephrotic syndrome with pulmonary embolism. Clin Nucl Med 2000;25:167–72.
  14. Burstein DM, et al. Membranous glomerulonephritis and malignancy. Am J Kidney Dis 1993;22:5–10.
  15. Lefaucheur C, et al. Membranous nephropathy and cancer:Epidemiologic evidence and determinants of high-risk cancer association. Kidney Int 2006;70:1510–7.
  16. Bjorneklett R, et al. Long-term risk of cancer in membranous nephropathy patients. Am J Kidney Dis 2007;50:396–03.
  17. Zeng CH, et al. Etiology and clinical characteristics of membranous nephropathy in Chinese patients. Am J Kidney Dis 2008;52:691–8.
  18. Yokoyama H, et al. Membranous nephropathy in Japan:Analysis of the Japan Renal Biopsy Registry(J-RBR). Clin Exp Nephrol 2012;16:557–63.
  19. Smith JD, et al. Reversible renal failure in the nephrotic syndrome. Am J Kidney Dis 1992;19:201–13.

IV. Treatment

1. Clinical questions for treatment
  1. *Minimal change nephrotic syndrome* · *focal segmental glomerulosclerosis*

CQ 1. Is oral steroid recommended for reducing urinary protein level and preventing the decline of renal function in minimal change nephrotic syndrome?

1. Gipson DS, et al. Pediatrics 2009;124:747–57. (Level 2)
2. Hodson EM, et al. Cochrane Database Syst Rev 2007:CD001533 (Level 2)
3. Black DA, et al. Br Med J 1970;3:421–6. (Level 2)
4. Coggins CH. Trans Am Clin Climatol Assoc 1986;97:18–26. (Level 2)
5. Mak SK, et al. Nephrol Dial Transplant 1996;11:2192–201. (Level 4)
6. Tse KC, et al. Nephrol Dial Transplant 2003;18:1316–20. (Level 4)
7. Palmer SC, et al. Cochrane Database Syst Rev 2008:CD001537 (Level 2)
8. Fujimoto S, et al. Am J Kidney Dis 1991;17:687–92. (Level 4)
9. Nakayama M, et al. Am J Kidney Dis 2002;39:503–12. (Level 4)
10. Nolasco F, et al. Kidney Int 1986;29:1215–23. (Level 4)
11. Al-Khader AA, et al. Clinical Nephrology 1979;11:26–30. (Level 4)
12. Imbasciati E, et al. Br Med J 1985;291:1305–8. (Level 2)
13. Fukudome K, et al. Nephrology 2012;17:263–8. (Level 4)
14. Tojo S, et al. Kidney and Dialysis. 1994;36:1053–64. (Level 2)
15. Nagasawa K, et al. Lupus 2005;14:385–90. (Level 3)

CQ 2. Is cyclosporine recommended for reducing urinary protein level and preventing the decline of renal function in minimal change nephrotic syndrome?

1. Hodson EM, et al. Cochrane Database Syst Rev 2010:CD003594 (Level 2)
2. Durkan AM, et al. Kidney Int 2001;59:1919–27. (Level 1)
3. Ponticelli C, et al. Nephrol Dial Transplant 1993;8:1326–32. (Level 2)
4. Niaudet P. Pediatr Nephrol 1992;6:1–3. (Level 2)
5. Ishikura K, et al. Kidney Int 2008;73:1167–73. (Level 2)
6. Eguchi A, et al. Nephrol Dial Transplant 2010;25:124–9. (Level 2)

CQ 3. Is steroid therapy recommended for reducing urinary protein and preventing the decline of renal function in focal segmental glomerulosclerosis?

1. Troyanov S, et al. J Am Soc Nephrol 2005;16:1061–8. (Level 4)
2. Goumenos DS, et al. Nephron Clin Pract 2006;104:c75–82. (Level 4)
3. Pei Y, et al. Am J Med 1987;82:938–44. (Level 4)
4. Stirling CM, et al. QJM 2005;98:443–9. (Level 4)
5. Korbet SM, et al. Am J Kidney Dis 1994;23:773–83. (Level 2)
6. Banfi G, et al. Clin Nephrol 1991;36:53–9. (Level 4)
7. Cattran DC, et al. Am J Kidney Dis 1998;32:72–9. (Level 4)
8. Rydel JJ, et al. Am J Kidney Dis 1995;25:534–42. (Level 4)
9. Hari P, et al. Pediatr Nephrol 2001;16:901–5. (Level 4)
10. Kirpekar R, et al. Am J Kidney Dis 2002;39:1143–52. (Level 4)
11. Chang JW, et al. Pediatr Int 2007;49:349–54. (Level 4)
12. Pena A, et al. Pediatr Nephrol 2007;22:1875–80. (Level 4)
13. Tune BM, et al. J Am Soc Nephrol 1997;8:824–32. (Level 4)

CQ 4. Is cyclosporine recommended for reducing urinary protein level and preventing the decline of renal function in focal segmental glomerulosclerosis?

1. Hodson EM, et al. Cochrane Database Syst Rev 2010(11):CD003594 (Level 1)
2. Durkan AM, et al. Kidney Int 2001;59:1919–27. (Level 1)
3. Ponticelli C, et al. Nephrol Dial Transplant 1993;8:1326–32. (Level 2)
4. Niaudet P, et al. Pediatr Nephrol 1992;6:1–3. (Level 2)
5. Ishikura K, et al. Kidney Int 2008;73:1167–73. (Level 2)
6. Ponticelli C, et al. Kidney Int 1993;43:1377–84. (Level 2)
7. Cattran DC, et al. Kidney Int 1999;56:2220–6. (Level 2)
8. Eguchi A, et al. Nephrol Dial Transplant 2010;25:124–9. (Level 2)
9. Braun N, et al. Cochrane Database Syst Rev 2008 (3):CD003233 (Level 1)
10. Heering P, et al. Am J Kidney Dis 2004;43:10–8. (Level 2)

CQ 5. Is the addition of immunosuppressive agents recommended for reducing urinary protein level or preventing the decline of renal function in frequently relapsing nephrotic syndrome?

1. Ponticelli C, et al. Nephrol Dial Transplant 1993;8:1326–32. (Level 2)
2. Mak SK, et al. Nephrol Dial Transplant 1996;11:2192–201. (Level 4)
3. Nolasco F, et al. Kidney Int 1986;29:1215–23. (Level 4)
4. Yoshioka K, et al. Kidney Int 2000;58:317–24. (Level 2)
5. Fujieda M, et al. Clin Nephrol 2008;69:179–84. (Level 5)
6. Doi T, et al. Clin Nephrol 2008;69:433–5. (Level 5)

CQ 6. Are immunosuppressive agents administered in conjunction with steroids recommended for reducing urinary protein and preventing the decline of renal function in steroid-resistant focal segmental glomerulosclerosis?

1. Braun N, et al. Cochrane Database Syst Rev 2008(3): CD003233 (Level 1)
2. Cattran DC, et al. Kidney Int 1999;56:2220–6. (Level 2)
3. Heering P, et al. Am J Kidney Dis 2004;43:10–8. (Level 2)
4. Gipson DS, et al. Kidney Int 2011;80:868–78. (Level 2)

2. *Membranous nephropathy*

CQ 7. Is no treatment or supportive treatment alone without immunosuppressive agents recommended for reducing urinary protein level and preventing the decline of renal function in membranous nephropathy with nephrotic syndrome?

1. Polanco N, et al. J Am Soc Nephrol 2010;21:697–704. (Level 4)
2. Gansevoort RT, et al. Nephrol Dial Transplant 1992;7(Suppl 1):91–6. (Level 3)
3. Praga M, et al. Am J Kidney Dis 1992;20:240–8. (Level 5)
4. Shiiki H, et al. Kidney Int 2004;65:1400–7. (Level 4)

CQ 8. Is steroid-alone treatment recommended for reducing urinary protein level and preventing the decline of renal function in membranous nephropathy?

1. Coggins CH, et al. N Engl J Med 1979;301:1301–6. (Level 2)
2. Cattran DC, et al. N Engl J Med 1989;320:210–5. (Level 2)
3. Cameron JS, et al. QJM 1990;74:133–56. (Level 2)
4. Shiiki H, et al. Kidney Int 2004;65:1400–7. (Level 4)
5. Ponticelli C, et al. N Engl J Med 1992;327:599–603. (Level 2)
6. Pahari DK, et al. J Assoc Physicians India 1993;41:350–1. (Level 3)
7. Cattran DC, et al. Kidney Int 2001;59:1484–90. (Level 2)

CQ 9. Is cyclosporine recommended for reducing urinary protein level and preventing the decline of renal function in membranous nephropathy?

1. Schieppati A, et al. Cochrane Database Syst Rev 2004(4):CD004293 (Level 2)
2. Perna A, et al. Am J Kidney Dis 2004;44:385–401. (Level 1)
3. Cattran DC, et al. Kidney Int 2001;59:1484–90. (Level 2)
4. Alexopoulos E, et al. Nephrol Dial Transplant 2006;21:3127–32. (Level 4)
5. Naumovic R, et al. Biomed Pharmacother 2011;65:105–10. (Level 2)
6. Satio T, et al. Clin Exp Nephrol, in press (Level 2)

CQ 10. Is mizoribine recommended for reducing urinary protein level and preventing the decline of renal function in membranous nephropathy?

1. Shibasaki T, et al. Clin Exp Nephrol 2004;8:117–26. (Level 3)

CQ 11. Are alkylating agents recommended for reducing urinary protein level and preventing the decline of renal function in membranous nephropathy?

1. Schieppati A, et al. Cochrane Database Syst Rev 2004(4):CD004293 (Level 2)
2. Perna A, et al. Am J Kidney Dis 2004;44:385–401. (Level 1)
3. Ponticelli C, et al. N Engl J Med 1992;327:599–603. (Level 2)
4. Falk RJ, et al. Ann Intern Med 1992;116:438–45. (Level 2)
5. Shiiki H, et al. Kidney Int 2004;65:1400–7. (Level 4)
6. Hofstra JM, et al. Nephrol Dial Transplant 2008;23:3534–8. (Level 4)
7. Naumovic R, et al. Biomed Pharmacother 2010;64:633–8. (Level 4)
8. Eriguchi M, et al. Nephrol Dial Transplant 2009;24:3082–8. (Level 1)
9. Bizzarri D, et al. Contrib Nephrol 1993;105:65–70. (Level 4)
10. Branten AJ, et al. Am J Kidney Dis 2007;50:248–56. (Level 3)
11. Ponticelli C, et al. J Am Soc Nephrol 1998;9:444–50. (Level 2)

CQ 12. Are conservative treatments recommended for reducing urinary protein level and preventing the decline of renal function in membranous nephropathy showing a non-nephrotic range of proteinuria?

1. Hladunewich MA, et al. Clin J Am Soc Nephrol 2009;4:1417–22. (Level 4)

3. *Membranoproliferative glomerulonephritis*

CQ 13. Is steroid treatment recommended for reducing urinary protein level and preventing the decline of renal function in idiopathic membranoproliferative glomerulonephritis showing nephrotic syndrome?

1. Tarshish P, et al. Pediatr Nephrol 1992;6:123–30. (Level 2)
2. McEnery PT. J Pediatr 1990;116:S109–14. (Level 4)
3. Warady BA, et al. J Pediatr 1985;107:702–7. (Level 4)
4. Emre S, et al. Acta Paediatr Jpn 1995;37:626–9. (Level 4)
5. Bergstein JM, et al. Pediatr Nephrol 1995;9:268–71. (Level 4)
6. Ford DM, et al. Kidney Int 1992;41:1606–12. (Level 4)
7. Donadio JV Jr, et al. Am J Kidney Dis 1989;14:445–51. (Level 5)

4. *Steroid treatment*

CQ 14. Is oral steroid treatment recommended during intervals between steroid pulse treatments (i.e., at days when no steroid pulse treatment is given)?

CQ 15. Is the increase of oral steroid doses or the change of administration routes recommended for patients with systemic edema?

1. Frey FJ, et al. Am J Kidney Dis 1984;3:339–48. (Level 4)
2. Bergrem H. Kidney Int 1983;23:876–81. (Level 4)

CQ 16. Is alternate-day administration as a means of steroid dose reduction effective for inhibiting the incidence of adverse effects?

1. Mak SK, et al. Nephrol Dial Transplant 1996;11:2192–201. (Level 4)
2. Waldman M, et al. Clin J Am Soc Nephrol 2007;2:445–53. (Level 4)
3. Carter ME, et al. Ann Rheum Dis 1972;31:379–83. (Level 4)
4. Kimura Y, et al. J Rheumatol 2000;27:2018–24. (Level 4)
5. Byron MA, et al. J R Soc Med 1983;76:452–7. (Level 4)

CQ 17. Is reducing the steroid dose compared with that of the first treatment recommended for the treatment of recurrent nephrotic syndrome?

1. Fujimoto S, et al. Am J Kidney Dis 1991;17:687–92. (Level 4)
2. Nakayama M, et al. Am J Kidney Dis 2002;39:503–12. (Level 4)
3. Takei T, et al. Clin Exp Nephrol 2007;11:214–7. (Level 4)
4. Imbasciati E, et al. Br Med J 1985;291:1305–8. (Level 2)

CQ 18. Is there a standard period for steroid maintenance therapy after nephrotic syndrome has remitted?

1. Huang JJ, et al. Am J Nephrol 2001;21:28–34. (Level 4)
2. Mak SK, et al. Nephrol Dial Transplant 1996;11:2192–201. (Level 4)
3. Waldman M, et al. Clin J Am Soc Nephrol 2007;2:445–53. (Level 4)
4. Korbet SM, et al. Am J Kidney Dis 1994;23:773–83. (Level 2)
5. Banfi G, et al. Clin Nephrol 1991;36:53–9. (Level 4)
6. Cattran DC, et al. Am J Kidney Dis 1998;32:72–9. (Level 4)
7. Rydel JJ, et al. Am J Kidney Dis 1995;25:534–42. (Level 4)
8. Tarshish P, et al. Pediatr Nephrol 1992;6:123–30. (Level 2)
9. McEnery PT. J Pediatr 1990;116:S109–14. (Level 4)
10. Warady BA, et al. J Pediatr 1985;107:702–7. (Level 4)
11. Bergstein JM, et al. Pediatr Nephrol 1995;9:268–71. (Level 4)

5. *Immunosuppressive agents not allowed by medical insurance (at the time of description of this guideline in 2013)*

CQ 19. Is rituximab recommended for reducing urinary protein level and preventing the decline of renal function in nephrotic syndrome?

1. Sugiura H, et al. Nephron Clin Pract 2011;117:c98–105. (Level 5)
2. Fernandez-Fresnedo G, et al. Clin J Am Soc Nephrol 2009;4:1317–23. (Level 4)
3. Ravani P, et al. Clin J Am Soc Nephrol 2011;6:1308–15. (Level 2)
4. Bomback AS, et al. Clin J Am Soc Nephrol 2009;4:734–44. (Level 5)
5. Segarra A, et al. Clin J Am Soc Nephrol 2009;4:1083–8. (Level 5)
6. Fervenza FC, et al. Clin J Am Soc Nephrol 2010;5:2188–98. (Level 5)

CQ 20. Is mycophenolate mofetil recommended for reducing urinary protein level and preventing the decline of renal function in nephrotic syndrome?

1. Lee YH, et al. Lupus 2010;19:703–10. (Level 2)
2. Zhu B, et al. Nephrol Dial Transplant 2007;22:1933–42. (Level 1)
3. Dorresteijn EM, et al. Pediatr Nephrol 2008;23:2013–20. (Level 2)
4. Ito S, et al. Pediatr Nephrol 2011;26:1823–8. (Level 3)
5. Senthil Nayagam L, et al. Nephrol Dial Transplant 2008;23:1926–30. (Level 2)
6. Dussol B, et al. Am J Kidney Dis 2008;52:699–705. (Level 2)
7. Branten AJ, et al. Am J Kidney Dis 2007;50:248–56. (Level 3)
8. Chan TM, et al. Nephrology (Carlton) 2007;12:576–81. (Level 2)

CQ 21. Is azathioprine recommended for reducing urinary protein level and preventing the decline of renal function in nephrotic syndrome?

1. Sharpstone P, et al. Br Med J 1969;2:535–9. (Level 2)
2. Cade R, et al. Arch Intern Med 1986;146:737–41. (Level 4)
3. Hiraoka M, et al. Pediatr Nephrol 2000;14:776–8. (Level 5)
4. Goumenos DS, et al. Nephron Clin Pract 2006;104:c75–82. (Level 4)
5. Abramowicz M, et al. Lancet 1970;(1 7654):959–61. (Level 2)
6. Adeniyi A, et al. Arch Dis Child 1979;54:204–7. (Level 2)
7. Habashy D, et al. Pediatr Nephrol 2003;18:906–12. (Level 2)
8. Colquitt JL, et al. Health Technol Assess 2007;11:iii–iv, ix–xi, 1–93. (Level 2)
9. Western Canadian Glomerulonephritis Study Group, et al. Can Med Assoc J 1976;115:1209–10. (Level 2)
10. Ahuja M, et al. Am J Kidney Dis 1999;34:521–9. (Level 2)
11. Goumenos DS, et al. Clin Nephrol 2006;65:317–23. (Level 3)
12. Brown JH, et al. Nephrol Dial Transplant 1998;13:443–8. (Level 4)
13. Williams PS, et al. Nephrol Dial Transplant 1989;4:181–6. (Level 4)
14. Naumovic R, et al. Biomed Pharmacother 2011;65:105–10. (Level 2)

6. *Nephrotic syndrome in the elderly*

CQ 22. Are immunosuppressive agents recommended for elderly patients with nephrotic syndrome?

1. Deegens JK, et al. Drugs Aging 2007;24:717–32. (Level 5)
2. Passerini P, et al. Nephrol Dial Transplant 1993;8:1321–5. (Level 3)
3. Bizzarri D, et al. Contrib Nephrol 1993;105:65–70. (Level 5)
4. Nolasco F, et al. Kidney Int 1986;29:1215–23.
5. Tse KC, et al. Nephrol Dial Transplant 2003;18:1316–20. (Level 4)
6. Al-Khader AA, et al. Clin Nephrol 1979;11:26–30. (Level 4)
7. Nagai R, et al. Clin Nephrol 1994;42:18–21. (Level 4)
8. Zent R, et al. Am J Kidney Dis 1997;29:200–6. (Level 4)
9. Branten AJ, et al. QJM 1998;91:359–66. (Level 4)
10. Ponticelli C, et al. J Am Soc Nephrol 1998;9:444–50. (Level 2)
11. Quaglia M, et al. Drugs 2009;69:1303–17. (Level 5)

7. *Adjunctive and supportive treatments*

CQ 23. Are renin-angiotensin system (RAS) inhibitors recommended for reducing urinary protein level in nephrotic syndrome?

1. The GISEN group. Lancet 1997;349:1857–63. (Level 2)
2. Polanco N, et al. J Am Soc Nephrol 2010;21:697–704. (Level 2)
3. Kosmadakis G, et al. Scand J Urol Nephrol 2010;44:251–6. (Level 2)
4. Giri S, et al. J Assoc Physicians India 2002;50:1245–9. (Level 2)
5. Usta M, et al. J Intern Med 2003;253:329–34. (Level 2)
6. Cheng J, et al. Int J Clin Pract 2009;63:880–8. (Level 1)
7. Kincaid-Smith P, et al. Nephrol Dial Transplant 2002;17:597–601. (Level 2)
8. Tomino Y, et al. J Nephrol 2009;22:224–31. (Level 4)
9. Nakamura T, et al. Am J Hypertens 2007;20:1195–201. (Level 2)
10. Parving HH, et al. N Engl J Med 2008;358:2433–46. (Level 2)
11. Navaneethan SD, et al. Clin J Am Soc Nephrol 2009;4:542–51. (Level 1)
12. Parving HH, et al. N Engl J Med 2012;367:2204–13. (Level 2)

CQ 24. Are diuretics recommended for reducing edema in nephrotic syndrome?

1. Nakahama H, et al. Nephron 1988;49:223–7. (Level 4)
2. Kapur G, et al. Clin J Am Soc Nephrol 2009;4:907–13. (Level 4)
3. Ostermann M, et al. Nephron Clin Pract 2007;107:c70–6. (Level 2)
4. Felker GM, et al. N Engl J Med 2011;364:797–805. (Level 2)
5. Fliser D, et al. Kidney Int 1999;55:629–34. (Level 4)
6. Akcicek F, et al. BMJ 1995;310:162–3. (Level 4)

CQ 25. Is albumin administration recommended to improve hypoalbuminemia in nephrotic syndrome?

1. Fliser D, et al. Kidney Int 1999;55:629–34. (Level 4)
2. Ghafari A, et al. Saudi J Kidney Dis Transpl 2011;22:471–5. (Level 4)
3. Na KY, et al. J Korean Med Sci 2001;16:448–54. (Level 2)
4. Dharmaraj R, et al. Pediatr Nephrol 2009;24:775–82. (Level 4)
5. Haws RM, et al. Pediatrics 1993;91:1142–6. (Level 4)
6. Yoshimura A, et al. Clin Nephrol 1992;37:109–14. (Level 4)

CQ 26. Are antiplatelet and anticoagulant agents recommended for reducing urinary protein level and preventing thrombosis in nephrotic syndrome?

1. Kamei K, et al. Clin J Am Soc Nephrol 2011;6:1301–7. (Level 2)
2. Taji Y, et al. Clin and Exp Nephrol 2006;10:268–73. (Level 1)
3. Nakamura T, et al. Nephron 2001;88:80–2. (Level 2)
4. Nakamura T, et al. Diabetes Care 2000;23:1168–71. (Level 2)
5. Liu XJ, et al. Intern Med 2011;50:2503–10. (Level 1)
6. Tojo S, et al. Contrib Nephrol 1978;9:111–27. (Level 4)
7. Zäuner I, et al. Nephrol Dial Transplant 1994;9:619–22. (Level 2)
8. Lilova MI, et al. Pediatr Nephrol 2000;15:74–8. (Level 4)
9. Sarasin FP, et al. Kidney Int 1994;45:578–85. (Level 4)

CQ 27. Are statins recommended to improve dyslipidemia and life prognosis in nephrotic syndrome?

1. Ordoñez JD, et al. Kidney Int 1993;44:638–42. (Level 4)
2. Valdivielso P, et al. Nephrology(Carlton) 2003;8:61–4. (Level 2)
3. Fried LF, et al. Kidney Int 2001;59:260–9. (Level 4)
4. Gheith OA, et al. Nephron 2002;91:612–9. (Level 2)
5. Sandhu S, et al. J Am Soc Nephrol 2006;17:2006–16. (Level 4)
6. Rayner BL, et al. Clin Nephrol 1996;46:219–24. (Level 2)
7. Dogra GK, et al. Kidney Int 2002;62:550–7. (Level 4)

CQ 28. Is ezetimibe recommended to improve lipid metabolism abnormalities and life prognosis in nephrotic syndrome?

1. Baigent C, et al. Lancet 2011;377:2181–92. (Level 2)
2. Nakamura T, et al. Pharmacol Res 2010;61:58–61. (Level 2)

CQ 29. Is low-density lipoprotein (LDL) apheresis recommended for reducing urinary protein levels in refractory nephrotic syndrome?

1. Tojo K, et al. Jpn J Nephrol 1988;30:1153–60. (Level 5)
2. Muso E, et al. Nephron 2001;89:408–15. (Level 4)
3. Muso E, et al. Clin Nephrol 2007;67:341–4. (Level 4)
4. Hattori M, et al. Am J Kidney Dis 2003;42:1121–30. (Level 5)

CQ 30. Is the extracorporeal ultrafiltration method (ECUM) recommended for refractory edema and ascites in nephrotic syndrome?

1. Smith DE, et al. J Pharm Sci 1985;74:603–7. (Level 5)
2. Keller E, et al. Clin Pharmacol Ther 1982;32:442–9. (Level 5)
3. Asaba H, et al. Acta Med Scand 1978;204:145–9. (Level 5)
4. Fauchald P, et al. Acta Med Scand 1985;217:127–31. (Level 5)

CQ 31. Is the trimethoprim-sulfamethoxazole combination recommended for preventing infectious disease during immunosuppressive therapy in nephrotic syndrome?

CQ 32. Is immunoglobulin supply recommended for preventing infectious disease in nephrotic syndrome?

1. Wu HM,et al. Cochrane Database Syst Rev 2012;4:CD003964. (Level 1)
2. Ogi M,et al. Am J Kidney Dis 1994;24:427–36. (Level 4)

CQ 33. Is treatment with antitubercular agents recommended for preventing tuberculous infection in nephrotic syndrome?

1. Gulati S, et al. Pediatr Nephrol 1995;9:431–4. (Level 4)
2. Gulati S, et al. Pediatr Nephrol 1997;11:695–8. (Level 4)
3. Currie AC, et al. Transplantation 2010;90:695–704. (Level 1)
4. Naqvi R, et al. Nephrol Dial Transplant 2010;25:634–7. (Level 2)

CQ 34. Is immunosuppressive therapy recommended for patients with hepatitis B-positive nephrotic syndrome?

8. *Lifestyle and dietary instruction*

CQ 35. Is the prevalence rate of cancer in patients with membranous nephropathy higher than that in the general population?

1. Bjørneklett R, et al. Am J Kidney Dis 2007;50:396–403. (Level 4)
2. Lefaucheur C, et al. Kidney Int 2006;70:1510–7. (Level 4)
3. Burstein DM, et al. Am J Kidney Dis 1993;22:5–10. (Level 5)

CQ 36. Is bed rest/exercise restriction recommended in nephrotic syndrome?

1. Fuiano G, et al. Am J Kidney Dis 2004;44:257–63. (Level 5)

CQ 37. Is vaccination recommended in patients with nephrotic syndrome during treatment with corticosteroids and immunosuppressive drugs?

1. Fuchshuber A, et al. Nephrol Dial Transplant 1996;11:468–73. (Level 4)
2. Collins AJ, et al. Am J Kidney Dis 2008;51:S1–320. (Level 4)

CQ 38. Are there any preventive measures against steroid-induced femoral head necrosis in nephrotic syndrome?

1. Nagasawa K, et al. Lupus 2006;15:354–7. (Level 3)
2. Ajmal M, et al. Orthop Clin North Am 2009;40:235–9. (Level 4)
3. Lai KA, et al. J Bone Joint Surg Am 2005;87:2155–9. (Level 2)
4. Wang CJ, et al. Arch Orthop Trauma Surg 2008;128:901–8. (Level 2)

CQ 39. Is the avoidance of mental stress recommended to prevent the onset and relapse of nephrotic syndrome?

1. Takahashi S, et al. J Jpn Pediatr Soc 1996;100:72–7. (Level 4)
2. Takahashi S, et al. Pediatr Nephrol 2007;22:232–6. (Level 4)

CQ 40. Is a fat-restricted diet recommended to improve dyslipidemia and life prognosis in nephrotic syndrome?

1. D’Amico G, et al. Clin Nephrol 1991;35:237–42. (Level 4)
2. D’Amico G, et al. Lancet 1992;339:1131–4. (Level 4)

2. Dietary instruction
  1. Rodriguez-Iturbe B, et al. Atrial natriuretic factor in theacute nephritic and nephrotic syndromes. Kidney Int 1990;38:512–7.
  2. Kaysen GA, et al. Effect of dietary protein intake on albumin homeostasis in nephrotic patients. Kidney Int 1986;29:572–7.
  3. D’Amico G, et al. Effect of dietary proteins and lipids in patients with membranous nephropathy and nephrotic syndrome. Clin Nephrol 1991;35:237–42.
  4. Walser M, et al. Treatment of nephrotic adults with a supplemented, very low-protein diet. Am J Kidney Dis 1996;28:354–64.
  5. D’Amico G, et al. Effect of vegetarian soy diet on hyperlipidaemia in nephrotic syndrome. Lancet 1992;339:1131–4.
  6. Kopple JD, et al. Effect of energy intake on nitrogen metabolism in nondialyzed patients with chronic renal failure. Kidney Int 1986;29:734–42.
  7. Kaysen GA, et al. Effect of dietary protein intake on albumin homeostasis in nephrotic patients. Kidney Int 1986;29:572–7.
  8. Maroni BJ, et al. Mechanisms permitting nephrotic patients to achieve nitrogen equilibrium with a protein-restricted diet. J Clin Invest 1997;99:2479–87.
  9. Lim VS, et al. Leucine turnover in patients with nephrotic syndrome:evidence suggesting body protein conservation. J Am Soc Nephrol 1998;9:1067–73.
  10. Fouque D, et al. A proposed nomenclature and diagnostic criteria for protein-energy wasting in acute and chronic kidney disease. Kidney Int 2008;73:391–8.
  11. Kendirli T, et al. Vitamin B6 deficiency presenting with low alanine aminotransferase in a critically ill child. Pediatrics Int 2009;51:597–9.
  12. Nishida M, et al. Wernicke’s encephalopathy in a patient with nephrotic syndrome. Eur J Pediatr 2008;168:731–4.
  13. Banerjee S, et al. Vitamin D in nephrotic syndrome remission:a case–control study. Pediatr Nephrol 2013;28:1983–9.
  14. El-Melegy NT, et al. Oxidative Modification of Low-Density Lipoprotein in Relation to Dyslipidemia and Oxidant Status in Children With Steroid Sensitive Nephrotic Syndrome. Pediatr Res 2008;63:404–9.

